# Cross-ancestry analyses identify new genetic loci associated with 25-hydroxyvitamin D

**DOI:** 10.1371/journal.pgen.1011033

**Published:** 2023-11-14

**Authors:** Xiaotong Wang, Valentin Hivert, Shiane Groot, Ying Wang, Loic Yengo, John J. McGrath, Kathryn E. Kemper, Peter M. Visscher, Naomi R. Wray, Joana A. Revez

**Affiliations:** 1 Institute for Molecular Bioscience, The University of Queensland, Brisbane, Queensland, Australia; 2 Analytic and Translational Genetics Unit, Massachusetts General Hospital, Boston, Massachusetts, United States of America; 3 Stanley Center for Psychiatric Research and Program in Medical and Population Genetics, Broad Institute of Harvard and MIT, Cambridge, Massachusetts, United States of America; 4 National Centre for Register-Based Research, Aarhus University, Aarhus V, Denmark; 5 Queensland Centre for Mental Health Research, The Park Centre for Mental Health, Brisbane, Queensland, Australia; 6 Queensland Brain Institute, The University of Queensland, Brisbane, Queensland, Australia; University of Pennsylvania, UNITED STATES

## Abstract

Vitamin D status–a complex trait influenced by environmental and genetic factors–is tightly associated with skin colour and ancestry. Yet very few studies have investigated the genetic underpinnings of vitamin D levels across diverse ancestries, and the ones that have, relied on small sample sizes, resulting in inconclusive results. Here, we conduct genome-wide association studies (GWAS) of 25 hydroxyvitamin D (25OHD)–the main circulating form of vitamin D–in 442,435 individuals from four broad genetically-determined ancestry groups represented in the UK Biobank: European (N = 421,867), South Asian (N = 9,983), African (N = 8,306) and East Asian (N = 2,279). We identify a new genetic determinant of 25OHD (rs146759773) in individuals of African ancestry, which was not detected in previous analysis of much larger European cohorts due to low minor allele frequency. We show genome-wide significant evidence of dominance effects in 25OHD that protect against vitamin D deficiency. Given that key events in the synthesis of 25OHD occur in the skin and are affected by pigmentation levels, we conduct GWAS of 25OHD stratified by skin colour and identify new associations. Lastly, we test the interaction between skin colour and variants associated with variance in 25OHD levels and identify two loci (rs10832254 and rs1352846) whose association with 25OHD differs in individuals of distinct complexions. Collectively, our results provide new insights into the complex relationship between 25OHD and skin colour and highlight the importance of diversity in genomic studies. Despite the much larger rates of vitamin D deficiency that we and others report for ancestry groups with dark skin (e.g., South Asian), our study highlights the importance of considering ancestral background and/or skin colour when assessing the implications of low vitamin D.

## Author summary

Vitamin D synthesis in the skin is inversely related to the level of skin pigmentation. This is reflected in lower vitamin D levels in dark-skin populations. Asides from environmental determinants, like sun exposure, there are also genetic factors that influence vitamin D levels. Yet, despite the intertwined relationship with skin colour, genetic studies of vitamin D have largely focused on white individuals of European ancestry. Here, we study the genetic determinants of vitamin D across four ancestry groups represented in the UK Biobank (European, African, South Asian, and East Asian). We show that genetic effects on the main circulating form of vitamin D are correlated in genetically-distant groups, suggesting that the biological mechanisms underlying its synthesis and metabolism are largely shared. Nevertheless, we identify a new association with a genetic variant only seen in African-ancestry individuals. We find new associations with genetic variants that influence both skin colour and vitamin D levels, and we report variants with different effects in individuals with different complexions. Our study highlights the importance of considering ancestral background and/or skin complexion when assessing vitamin D status and its implications. This is relevant given the large numbers of ongoing clinical trials using vitamin D as intervention.

## Introduction

Vitamin D deficiency–a condition associated with several adverse health outcomes [1–6]–is tightly associated with ancestral background and skin colour. In populations originating near the tropics, where ultraviolet radiation (UVR) is more intense, dark skin is thought to have been selected due to factors related to skin cancer and/or for protection of folic acid synthesis pathways (crucial for cell division), despite detrimental to vitamin D biosynthesis [7–9]. Conversely, as populations migrated away from the tropics, into regions with lower UVR, need for vitamin D prevailed as the selective force that drove the evolution of facultative pigmentation (tanning), and depigmentation [8]. The effect of these opposing selective pressures on skin colour is reflected in higher vitamin D deficiency rates in individuals with African and Asian ancestries compared to those of European descent, when measured either at the same or different latitudes [10–13].

Asides from the strong influence of environmental factors, such as sun exposure related to latitude, season, behaviour (i.e. outdoor activity) and nutritional intake, genetic factors have also been shown to associate with vitamin D levels. Specifically, genome-wide association studies (GWAS) have been key in identifying associated genetic loci [14–16]. In a recent study of 417,580 individuals from the UK Biobank (UKB), we estimated the heritability of vitamin D at 0.32 (*s*.*e*. = 0.01), with the SNP-based heritability (variation attributed to common [minor allele frequency [MAF] > 1%) variants) explaining 40% of the heritability [14]. Our GWAS identified 143 independent loci associated with vitamin D, implicating genes involved in lipid and lipoprotein metabolism, dermal tissue properties, and the sulphonation and glucuronidation of vitamin D. Yet, the genes involved in skin properties were not related to skin colour/pigmentation. Notably, like the vast majority of GWAS conducted to date [17,18], our study was restricted to individuals of European ancestry–the population with largest representation in the UKB. Indeed, very few studies have investigated the genetic underpinnings of vitamin D in other ancestry groups [19], and the ones that have, relied on small sample sizes, resulting in inconclusive results.

Genetic studies in populations of non-European ancestry are critical to gain a better understanding of the genetic architecture of traits like skin colour and vitamin D. Despite being under-represented in genomic studies, non-European populations are known to contribute disproportionately to the overall number of identified loci [18]. Notably, compared to populations with dark skin colour, those with light skin colour have significantly less variability in skin pigmentation than expected by chance, suggesting that there is less genetic variance and/or heterogeneity in populations with light skin colour [20], i.e., the European-ancestry populations that dominate GWAS. This is particularly relevant for vitamin D biology given the intertwined relationship between the two traits. Furthermore, the differences in genetic architecture (e.g., differences in patterns of linkage disequilibrium (LD) or frequencies of risk variants) in non-European populations can be leveraged to dissect loci that are identified in individuals of European ancestry, but with complex LD structures that impede fine mapping.

Similarly, fine population structure within a single ancestry group is likely to also account for part of the variation in vitamin D levels, as is the case for variation in skin colour, which occurs not only between population of different continents but also within the same continent [20,21]. The standard paradigm within a single-ancestry GWAS is to include within-ancestry principal components (wPCs) derived from genetic data from that specific ancestry as covariates in analyses. These are included to capture subtle population stratification that may be confounded with the trait of interest. Yet, this conservative approach risks removing real genetic variation in order to combat potential non-genetic confounding factors that could be associated with the genetic wPCs. We hypothesised that for vitamin D variation, on balance, inclusion of genetic wPCs would exclude real genetic signal because we predicted (rightly) that wPCs would be associated with skin characteristics (despite being computed in a specific ancestry group), which in turn are associated with vitamin D biosynthesis. Indeed, genetic scores for skin pigmentation have previously been shown to associate with 25OHD levels in Europeans and self-reported African Americans [22–24]. Yet, the samples in those studies were generally small and had limited statistical power, resulting in associations with inconsistent direction of effects.

Another analytical limitation in most GWAS conducted to date is that traits are typically only studied under an additive model. Indeed, empirical and theoretical evidence shows that the contribution from additive effects to most quantitative traits is much larger than the contribution from non-linear effects [25]. Nonetheless, some traits, like those involving pigmentation, are notorious examples of phenotypes with dominant and/or recessive patterns of inheritance [26,27], and have been proposed as candidate traits that will more likely benefit from modelling of non-linear effects in genetic prediction [17]. Given the direct relationship between skin colour and vitamin D, it is possible that such effects are also reflected in the genetic architecture of vitamin D.

**Fig 1:**
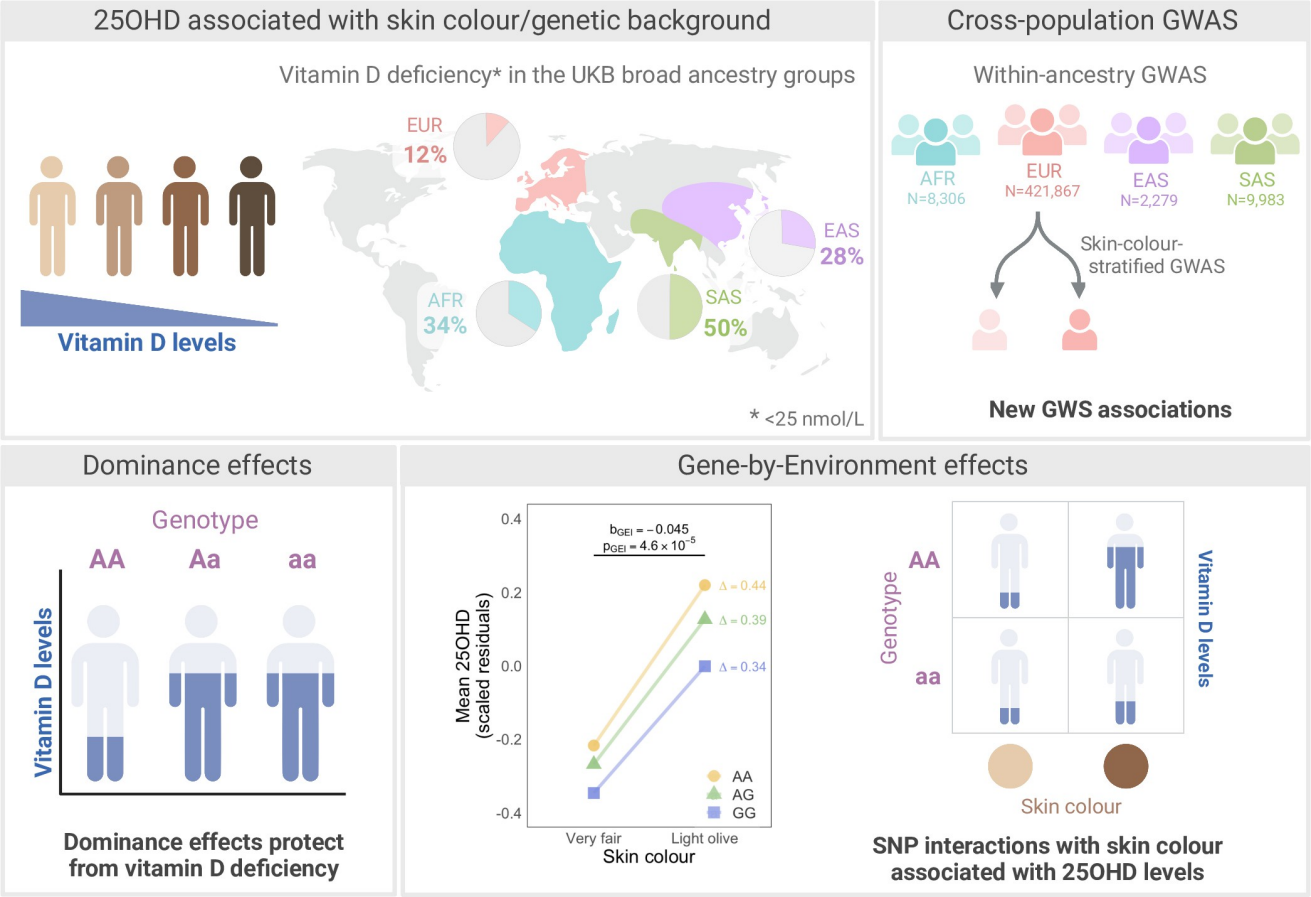
Study overview. Four broad genetically-defined ancestry groups represented in the UK Biobank (UKB) are analysed, namely European (EUR), African (AFR), South Asian (SAS), and East Asian (EAS). Observational analyses show that the prevalence of vitamin D “deficiency” (defined as < 25 nmol/L of 25 hydroxyvitamin D (25OHD)) is higher in groups with darker skin (top left panel). Within-ancestry genome-wide association studies (GWAS), including skin-colour-stratified GWAS in EUR, identify new loci associated with 25OHD at genome-wide significant (GWS) level (top right panel). A genome-wide dominance association study identifies loci with dominance patterns of inheritance that associate with higher 25OHD levels independently of additive effects (bottom left panel). Lastly, single nucleotide polymorphisms (SNPs) with different effects on 25OHD levels across individuals of different skin colour are identified in analysis of gene-by-environment interaction effects (bottom right panel). Created with BioRender.com.

Here, we leverage data from four genetically-defined ancestry groups represented in the UK Biobank (European, South Asian, African, and East Asian) to study the genetic architecture of 25-hydroxyvitamin D (25OHD)–the main circulating form of vitamin D. We explore (1) the genetic architecture of 25OHD in populations with different skin colour, looking at additive and dominance effects, (2) how ancestry-specific LD patterns can provide additional information on 25OHD-associated loci, (3) the effect of correcting for fine-population structure on the identification of 25OHD-associated loci, and (4) gene-environment interactions (**Fig 1**).

## Results

### 25OHD across populations

In total, 442,435 individuals (~90% of UKB participants, as of May 2023) had 25OHD measurements (data field 30890) available at the initial assessment visit. Of these, 421,867 were classified as European (EUR), 9,983 as South Asian (SAS), 8,306 as African (AFR), and 2,279 as East Asian (EAS). Assignment to the broad ancestry groups is described elsewhere [28] and in more detail in the Methods. Briefly, principal components (PCs) were computed for 2,000 participants of the 1000 genomes project (1KGP) from four superpopulations (EUR, SAS, AFR and EAS) [29]. Then, UKB participants were projected onto the 1KGP PCs and assigned to the closest ancestry based on the first three PCs.

The distribution of 25OHD levels was right skewed in all populations (**Fig A in S1 Appendix**). In line with previous observations [30,31], mean 25OHD levels were significantly higher (Wilcoxon Test *P* < 9.6 x 10^−296^) in EUR compared to the other ancestries (**Table A in S2 Appendix**), and the prevalence of vitamin D deficiency (defined as < 25 nmol/L of 25OHD) was higher in ancestry groups with darker skin (**Fig 1**). Assessment month was the top predictor of 25OHD levels, explaining 15%, 3.6%, 4.5%, and 9.0% of the variability in EUR, SAS, AFR, and EAS, respectively. The difference in variance explained by month of assessment across the ancestry groups (discussed in more detail in **Note A in S1 Appendix**) is consistent with a reduced effect of sunlight in populations with darker skin. Other variables that individually associated with 25OHD levels included within-ancestry PCs (see Methods; **Fig D in S1 Appendix**), supplement intake, assessment centre, age, use of sun protection, BMI, sex, genotyping batch, and time spent outdoors in summer, with some variation across ancestry groups (**Table D in S2 Appendix**). For example, assessment centre associated with 252OHD levels in all groups except EAS, and genotyping batch only associated with 25OHD levels in EUR. In a multiple regression fitting all variables, some of the individual effects of the predictor variables tested were no longer significant. For example, assessment centre no longer associated (P > 0.05/ 20 variables tested = 0.0025) with 25OHD in any of the non-EUR groups (**Table E in S2 Appendix**).

### Heritability and SNP-based heritability

Heritability estimates of 25OHD for proxy-pedigree relationships (coefficient of relationship [π] > 0.05) were lower in SAS than in EUR and AFR (${\hat{h}}_{SAS}^{2}=0.13,\;{\hat{h}}_{EUR}^{2}=0.32,\;{\hat{h}}_{AFR}^{2}=0.31;\;P_{SAS-EUR}=0.009,\;P_{SAS-AFR}=0.044$, Z-test difference between two estimates, H_0_: difference = 0; **Table F in S2 Appendix**), but noting the larger standard errors associated with the estimates in non-EUR individuals (*s*.*e*._*SAS*_ = 0.07, *s*.*e*._*EUR*_ = 0.01, *s*.*e*._*AFR*_ = 0.05), which reflect smaller sample sizes. SNP-based heritability estimates (${\hat{h}}_{SNP}^{2}$) from unrelated (π < 0.05) individuals suggested a larger contribution from common (MAF > 0.01) variants in AFR than in EUR (${\hat{h}}_{SNP-AFR}^{2}=0.23,\;s.e.=0.05$; and ${\hat{h}}_{SNP-EUR}^{2}=0.13,\;s.e.=0.009$; Z-test difference between two estimates *P* = 0.0478, H_0_: difference = 0; **Table F in S2 Appendix**).

### GWAS

Genetic variants with MAF > 0.002 (M_SAS_ = 14,396,495; M_AFR_ = 23,616,208; M_EAS_ = 11,732,894) were tested for association with 25OHD in each ancestry group, including a common set of 8,132,009 variants tested in all three groups (i.e., variants with MAF > 0.002 in the three groups) (**Figs E and F in S1 Appendix**). There were no genome-wide significant (GWS; *P* < 5 x 10^−8^) associations identified in EAS. Using conditional and joint analyses (GCTA–COJO [32], see Methods), we found one independent signal in SAS (in chromosome 4), and two in AFR (one in chromosome 2 and one in chromosome 4; **Table G in S2 Appendix; Fig E in S1 Appendix**). The same results were seen when analyses were further corrected for use of sun protection and/or time spent outdoors, depending on evidence of association with 25OHD in individual ancestry groups (**Note B in S1 Appendix**). The SNPs prioritised by COJO on chromosome 4 were in LD (r^2^_SAS_ = 0.92 and r^2^_AFR_ = 0.15) and replicate an association previously identified in EUR [14]. The SNP prioritised on chromosome 2 (rs146759773, MAF_AFR_ = 0.014) in AFR is an intronic variant within the protein kinase *PRKD3* that was rare in other ancestry groups (MAF < 0.002, hence not tested). There were no GWS associations in a 1-Mb window centred on that SNP in EUR (**Fig G in S1 Appendix**, panel B). To gain further insight into this association in the larger EUR cohort, we imputed gene expression of *PRKD3* and eight other genes in the region of association (i.e., within 500-Kb from rs146759773; see **Methods**), using gene expression from sun-exposed skin from the GTEx [33]. There was no association between genetically-predicted expression of *PRKD3* and 25OHD levels (b = -0.018, P = 0.16). However, four other genes in the region of association had genetically-predicted expression that associated with 25OHD levels in the EUR cohort at a Bonferroni-corrected threshold of *P* < 0.0056 (0.05/9 genes tested; **Table H in S2 Appendix**). Of these, *NDUFAF7*, *HEATR5B*, and *EIF2AK2* also had a nominally significant (*P* < 0.05) gene-based association with 25OHD (MAGMA results from Revez et al. 2020 [14]).

To assess the variability in genetic architecture of 25OHD across different ancestry groups, we compared associations identified in EUR with those assessed in AFR, a group with evidence of larger genetic diversity [34]. Specifically, we selected 143 variants previously shown to independently associate with 25OHD in EUR [14] and compared their effect size estimates with those estimated in our AFR cohort (**Table I in S2 Appendix**). Overall, there was a positive correlation (${\hat{r}}_{b}=0.62,\;s.e.=0.36$; see Methods) between effect sizes (**Fig H in S1 Appendix**, panel A) and variants with largest difference in effect size had similar allele frequencies (**Fig H in S1 Appendix**, panel B**; Table J in S2 Appendix**). The variant with the largest effect size difference (rs116970203; ${\hat{\beta }}_{EUR}=-0.377,\;{s.e.}_{EUR}=0.006,\;P_{EUR}=0;{\hat{\beta }}_{AFR}=0.054,\;{s.e.}_{AFR}=0.045,\;P_{AFR}=0.234$) had the same frequency in EUR and AFR (MAF = 0.03). To assess whether the association observed in EUR was driven by an associated causal variant, we identified variants in LD with rs116970203. The SNP was in perfect LD (LD r^2^ = 1) with rs117913124 in EUR, but not AFR (LD r^2^ = 0.05; **Fig I in S1 Appendix**). Interestingly, rs117913124 had similar effect size in both EUR and AFR (${\hat{\beta }}_{EUR}=-0.377,\;s.e.=0.006;\;{\hat{\beta }}_{AFR}=-0.305,\;s.e.=0.155$) (**Table J in S2 Appendix**), suggesting that the association observed in EUR was driven by rs117913124 or another variant in LD with it.

### Dominance GWAS

Pigmentation traits, such as hair and eye colour, are well known to exhibit dominance effects [35]. Given the relationship between pigmentation and vitamin D, we sought to determine whether non-additive genetic effects also contribute to the genetic architecture of vitamin D. Specifically, we conducted a dominance GWAS in individuals of European ancestry. Of 8,546,068 variants with MAF > 0.01, five variants (four in chromosome 11 and one in chromosome 16) were found to have GWS dominance effects (**Table K in S2 Appendix**, panel a**; Fig J in S1 Appendix**). The four variants on chromosome 11 were all less than 50 Kb away from each other but we confirmed through conditional analyses that they reflect two independent signals (**Fig K in S1 Appendix**; **Table K in S2 Appendix**, panel b). The first includes two variants in high LD (r^2^ = 1; rs1037378 and rs10766194) that are expression quantitative trait loci (eQTLs) for *COPB1*. The second includes two variants that are also in high LD with each other (r^2^ = 1; rs116970203 and rs117913124) but in low LD (r^2^ = 0.02) with the first two variants (**Table K in S2 Appendix**, panel a). The latter are eQTLs for *CYP2R1*, the gene that codes for the enzyme responsible for the start of vitamin D metabolism. Lastly, the dominance QTL in chromosome 16 was an eQTL and a stop-gained variant in *SDR42E1*, a gene overexpressed in skin [36] and predicted to be involved in steroid biosynthesis–relevant, since vitamin D is technically a steroid hormone produced endogenously in response to UVR exposure. Of note, this was the only dominance QTL with nominally significant additive (*P* = 2.5 x 10^−4^) [37] and dominance (*P* = 1.2 x 10^−3^) [35] effects on skin colour.

### Skin-colour-stratified GWAS

**Fig 2:**
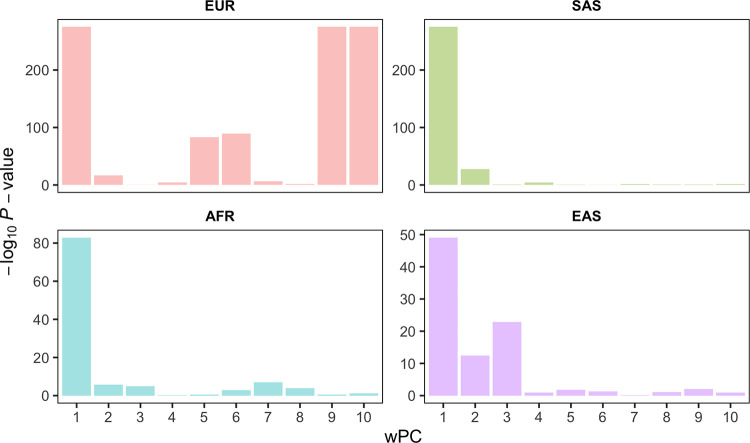
Association between PCs computed within ancestry group and skin colour. The -log_10_ *P*-value (y axis) of association between self-reported skin colour (UKB field 1717) and the first 10 within-ancestry principal components (wPCs). Association results are from a linear model fitting the 10 wPCs jointly. Together, the 10 wPCs explained 5%, 15%, 6%, and 18% of the variance in self-reported skin colour in EUR, SAS, AFR, and EAS, respectively. Statistics are presented in **Table L in S2 Appendix**. NB: since wPCs were computed within each ancestry group, results should not be compared across groups.

GWAS are typically conducted within single genetically-defined ancestry groups and use within-ancestry principal components (wPCs) to control for fine population structure. While critical to control for inflated type I error rate, this approach is also more likely to miss associations with traits that are ancestry-related if the wPCs capture those signals. That is likely the case for traits such as skin colour, which varies both between and within populations [20]. To assess if the with-ancestry PCs fitted in our GWAS (see Methods) captured variation in skin colour, we first tested the association between wPCs and self-reported skin colour in each of the ancestry groups represented in the UKB. We found significant associations between 3–5 of the first 10 ancestry wPCs and skin colour in each of the cohorts studied (**Table L in S2 Appendix**; **Fig 2**).

**Fig 3:**
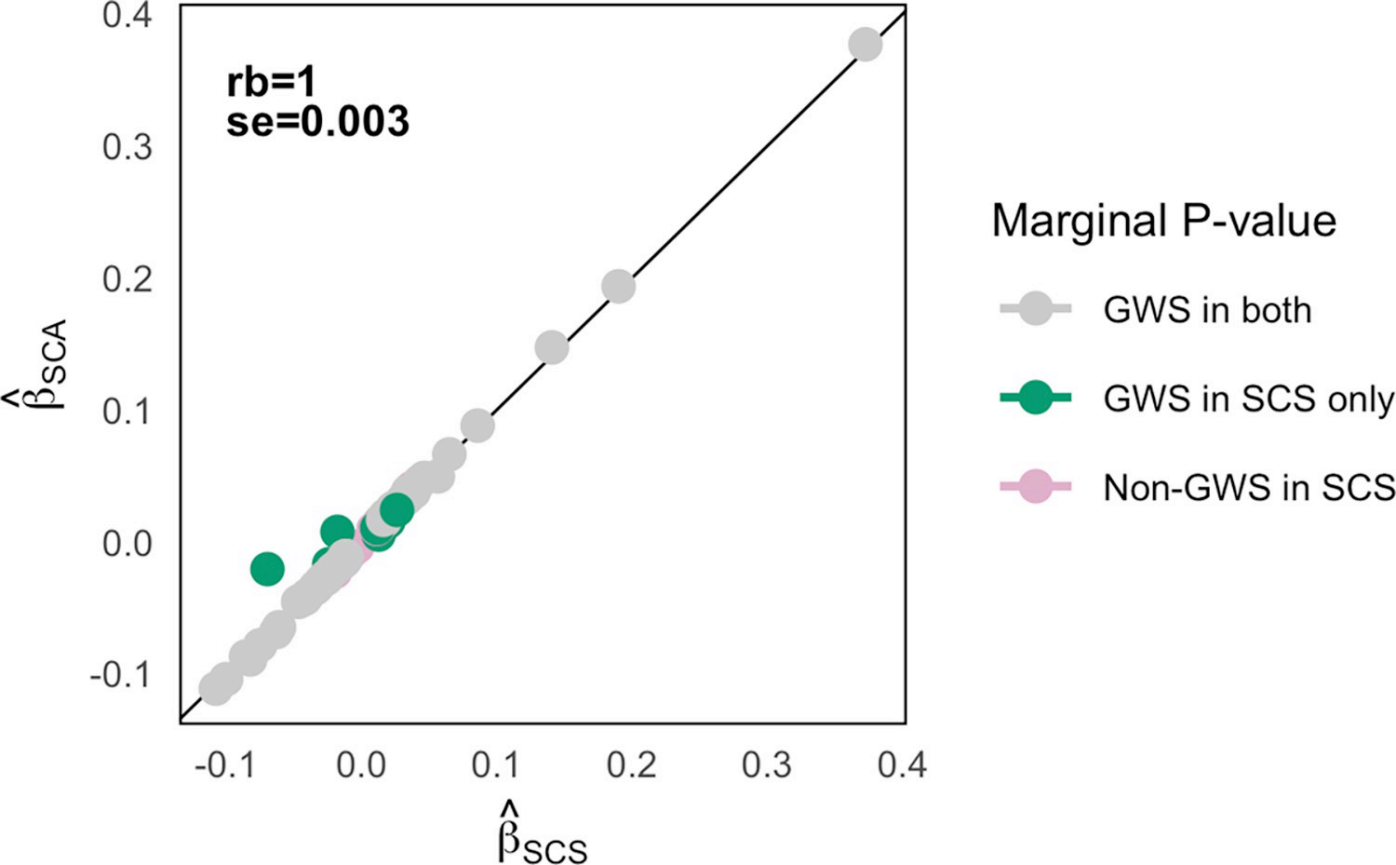
Comparison of effect size estimates of independent associations with RINT 25OHD in skin-colour-agnostic and skin-colour-stratified GWAS. Dots and bars represent the effect size estimates, and respective standard errors, in the skin-colour-agnostic (SCA) GWAS[14] (y axis) and in the skin-colour-stratified (SCS) meta-analysis (x axis) for 139 loci that independently (as assessed by conditional and joint analysis implemented in GCTA-COJO [32]) associated with 25OHD in the stratified analysis. Grey dots (N = 110) represent associations that were genome-wide significant (GWS) in both analyses. Green (N = 17) and pink (N = 12) dots represent associations that were, or were not, GWS in the SCS meta-analysis, respectively. rb is the correlation between effect size estimated adjusted for sampling error in both analyses and se is its standard error (see Methods).

We also conducted a number of analyses to assess a causal relationship between skin colour and vitamin D (see **Note C in S1 Appendix**). These analyses highlighted the complex relationship between skin colour, time spent outdoors, use of sunscreen and vitamin D levels. Given the intertwined relationship between skin colour and vitamin D levels, we then assessed if variants that are associated with 25OHD through skin colour were missed by adjusting for PCs. Specifically, we subset the European cohort (the group with largest representation in the UKB) into those self-reporting very fair or fair skin (N = 324,105) and those reporting olive or brown skin (N = 85,549). We opted to analyse two groups to maximise the contrast in pigmentation and minimise possible differences in people’s perception of skin colour (see Methods). Then, we conducted GWAS in both groups and meta-analysed the results. This approach adjusted for both mean and variance differences (**Table O in S2 Appendix**; **Fig L in S1 Appendix**) between the two skin colour groups. A total of 8,546,069 genetic variants with MAF > 0.01 in the light and dark skin groups were tested for association with 25OHD. Of those, 139 represented independent GWS associations with 25OHD, as defined by conditional and joint analysis (GCTA-COJO [32]; **Table P in S2 Appendix**; **Figs M** and **N in S1 Appendix**). There was a strong correlation in effect size between independent associations identified in the skin-colour-stratified GWAS and in the full skin-colour-agnostic GWAS (${\hat{r}}_{b}=1.0,\;s.e.=0.003$; See Methods; **Fig 3**), suggesting that the stratified approach reduced residual variance only. Still, 17 loci that independently associated with 25OHD at GWS level in the meta-analysis of the skin-colour-stratified GWAS were of specific interest. These were not detected at GWS level in the skin-colour-agnostic GWAS (henceforth referred to as ‘new loci’; **Table 1**). Closer inspection showed that three of these SNPs (rs10330, rs34650643, rs4841132) were within 1 Mb of variants that previously associated with 25OHD at GWS level in the skin-colour-agnostic GWAS [14], although the LD with those variants was low (r2 < 0.31). A key question was to determine if the new loci were statistical artifacts or if they are relevant to vitamin D biology. Among the new loci, four had no suggestive evidence (*P* > 10^−6^) of association with 25OHD in the skin-colour-agnostic GWAS (**Table 1**). The first (rs16891982) was a missense variant in *SLC45A2*, a gene that encodes a protein that mediates melanin synthesis (**Table Q in S2 Appendix**), and it was strongly associated with 25OHD levels in individuals of dark skin colour (*P* = 3 x 10^−25^; **Table P in S2 Appendix**). The second (rs12913832) was an intronic variant and an eQTL in whole blood for *HERC2* [36]*–*a gene harbouring variants associated with eye, skin, and hair pigmentation [37]. The third (rs35749740) was a variant associated with dermatological traits such as use of UV protection and skin and hair colour [37]. The fourth was an intronic variant in *RMDN2*. Sensitivity analyses showed that these new associations were unlikely to be driven by differences in the analytical approach used in the stratified and skin-colour-agnostic GWAS (**Note D in S1 Appendix**), but suggest that SNPs that were already near the GWS threshold (5 x 10^−8^ < *P* < 1 x 10^−6^) in the original unstratified GWAS are unlikely to be effects that were missed due to fitting PCs.

**Table 1:** 17 new loci identified to associate with 25OHD in meta-analysis of skin-colour-stratified GWAS.

				Meta-analysis of SCS GWAS					Full SCA GWAS				
CHR	SNP	BP	A1	Freq	BETA	SE	P	N	Freq	BETA	SE	P	N
5	rs16891982	33951693	C	0.05	-0.070	0.0061	7.2E-30	405,657	0.02	-0.021	0.0064	0.001	417,580
15	rs12913832	28365618	A	0.23	-0.018	0.0025	3.3E-13	405,657	0.22	0.007	0.0024	0.002	417,580
8	rs4841132	9183596	A	0.09	-0.024	0.0035	5.2E-12	405,657	0.09	-0.017	0.0035	7.2E-07	417,580
11	rs521765	89085801	C	0.87	0.020	0.0031	2.0E-10	389,159	0.87	0.015	0.0031	5.7E-07	400,808
9	rs34650643	107631312	G	0.80	-0.015	0.0025	2.2E-09	401,598	0.80	-0.013	0.0025	1.1E-07	413,402
19	19:4331940_AAAAG_A	4331940	AAAAG	0.66	0.012	0.0021	8.2E-09	393,994	0.66	0.011	0.0021	2.2E-07	405,784
1	rs35285316	34691983	C	0.83	-0.015	0.0027	1.9E-08	405,657	0.83	-0.014	0.0027	1.7E-07	417,580
15	rs4426330	90702129	A	0.77	-0.013	0.0024	2.4E-08	393,878	0.77	-0.012	0.0024	2.3E-07	405,581
6	rs742493	40998167	T	0.89	-0.018	0.0032	2.5E-08	405,657	0.89	-0.017	0.0032	1.5E-07	417,580
7	rs7790121	82723089	A	0.60	0.011	0.0021	2.9E-08	400,510	0.60	0.010	0.0020	4.4E-07	412,318
19	rs10330	19017862	G	0.87	0.017	0.0030	3.0E-08	405,657	0.87	0.015	0.0030	7.2E-07	417,580
4	rs576508629	44183941	C	0.95	0.026	0.0047	3.4E-08	403,696	0.95	0.025	0.0047	9.3E-08	415,563
16	rs35749740	90114833	T	0.71	0.012	0.0023	3.8E-08	385,999	0.71	0.005	0.0022	0.019	397,469
2	rs35270497	38259872	C	0.82	-0.015	0.0027	4.4E-08	398,987	0.82	-0.013	0.0026	5.9E-07	410,747
12	rs2306390	58002599	C	0.74	0.013	0.0023	4.6E-08	405,657	0.74	0.011	0.0023	6.1E-07	417,580
5	rs6887019	76630789	T	0.56	0.011	0.0020	4.7E-08	401,188	0.56	0.010	0.0020	7.9E-07	413,150
6	rs9501117	31398591	A	0.95	0.026	0.0048	4.8E-08	402,080	0.95	0.024	0.0047	4.0E-07	413,930

Marginal association results in the meta-analysis of skin-colour-stratified (SCS) GWAS and in the full skin-colour-agnostic (SCA) GWAS (Revez et al. 2020; PMID 32242144) for variants that independently (as assessed by conditional and joint analysis implemented in GCTA-COJO) associated with 25OHD at GWS level in the meta-analysis, but not the full SCA GWAS. Variants highlighted in yellow had no suggestive evidence (P > 10^−6^) of association in the SCA GWAS. SNP annotations are provided in **Table Q in S2 Appendix**. Columns are: CHR, chromosome; SNP, SNP rs ID; BP, physical position; A1, the effect allele; Freq, frequency of A1; BETA, effect size of A1; SE, standard error of the BETA; P, P-value; N, sample size

Of the 142 independent autosomal associations identified in the original skin-colour-agnostic GWAS [14], 19 were no longer GWS in the skin-colour-stratified analysis (henceforth referred to as ‘lost loci’; **Table S in S2 Appendix; Fig O in S1 Appendix**). Of those, one (rs138385079) was in LD (0.43 < r^2^ < 0.66) and within 1 Mb of variants that associated with 25OHD at GWS level in the skin-colour-stratified GWAS, suggesting that the association at that locus was detected in the stratified analysis. Under the assumption that the effect size estimated in the skin-colour-agnostic GWAS was the true effect size, power analyses showed that the power to identify the effect of the remaining 18 loci was the lowest of all associations identified (**Fig P in S1 Appendix**). Importantly, while the new loci were enriched for associations with pigmentation traits (five out of the 13 new loci that had GWAS results for association with pigmentation traits; empirical *P* = 0.0002; **Note E in S1 Appendix**; **Fig Q in S1 Appendix**; **Table U in S2 Appendix**), the 19 lost SNPs were not (< 1 out of the 14 lost loci that had GWAS results for association with pigmentation traits; empirical *P* = 0.75).

### Gene-environment interactions

To assess if there are genetic factors with different effects on 25OHD levels across individuals with different skin colour, we conducted a gene-environment interaction (GEI) analysis. Specifically, since GEIs can result in differences in variance between individuals with different genotypes [38], we assessed if self-reported skin colour interacted with vQTLs for 25OHD (i.e. variants associated with variance in 25OHD levels) [14]. Of 25 previously identified 25OHD vQTLs, two (rs10832254 and rs1352846) showed a significant (*P* < 0.002) interaction with skin colour (**Table V in S2 Appendix**). Specifically, in individuals homozygous for the rs10832254:A allele, 25OHD levels were 0.43 s.d. (standard deviation units; approximately 8.1 nmol/L) larger if they had light olive skin compared to very fair skin. However, with each additional copy of the rs10832254:G allele, the difference in 25OHD levels between the two skin colour groups was 0.045 s.d. lower (**Fig R in S1 Appendix**; **Table W in S2 Appendix**). That is, the difference in 25OHD levels between individuals with very fair and olive skin was 0.39 s.d. if they were heterozygous rs10832254:AG, and 0.34 s.d. (approximately 6.4 nmol/L) if they were homozygous rs10832254:GG. Similarly, 25OHD levels were lower in individuals of very fair skin compared to those fair and light olive skin, but that difference was attenuated with each additional copy of the rs1352846:G allele (**Fig R in S1 Appendix**; **Table W in S2 Appendix**). Of the 25 vQTLs previously identified, five (including rs10832254, but not rs1352846) showed a significant interaction with the season at which 25OHD levels were measured [14].

We conducted two GWAS with interaction terms for skin colour contrasting the two pigmentation groups that showed a significant interaction with a vQTL (i.e., very fair vs. fair skin, and very fair vs. light olive skin). There were no interactions reaching the genome-wide significance threshold of 5 x 10^−8^ in either GWAS. There was one suggestive (P <1 x 10^−6^) GEI when testing the interaction with very fair vs. fair skin, and three when testing the interaction with very fair vs. light olive skin (**Table X in S2 Appendix**). Of these, one (rs12271890) was in moderate LD (r^2^ = 0.4) with the vQTL (rs10832254) with which the interaction was first detected.

## Discussion

In this study, we leverage genetic differences across four broad ancestry groups represented in the UKB and the fine within-population structure to gain new insights into the genetic architecture of 25OHD. We observed a lower heritability of 25OHD in SAS compared to EUR and AFR. Skin colour is likely to contribute to these differences, but other environmental exposures and/or behavioural/cultural differences between the groups may also explain heritability differences across ancestry groups. For example, a longitudinal study comparing 25OHD levels in South Asian and Caucasian women living in the UK found that South Asian women had significantly lower weekly UVB exposure than Caucasian women [39]. Thus, behavioural and/or cultural differences may contribute to the differences in 25OHD levels and, consequently, 25OHD heritability estimates. This is also seen with differences in heritability in individuals assessed in summer vs. winter months, with lower heritability estimates seen in winter months, when there is lower UV exposure [14,40]. In terms of SNP-based heritability, AFR had a larger estimate than EUR, suggesting a larger contribution from common (MAF > 0.01) variants in AFR than in EUR. This is in line with the larger genetic diversity observed in AFR [34,41], but the difference in SNP-based heritability estimates was only marginally significant (*P* = 0.048).

Our GWAS in non-European individuals replicated a well-known association identified in Europeans on chromosome 4, implicating *GC*, the gene that codes to the protein responsible for transporting vitamin D to its target tissues (vitamin D binding protein, or DBP). DBP is a highly polymorphic protein and major variants (including rs7041 and rs4588 –two missense variants that determine key isoforms of DBP) have marked differences in allele frequency across different latitudes [29,42]. The variants identified in our SAS and AFR GWAS (rs17467825 and rs1352846, respectively) are in high LD (r^2^ > 0.90) with rs4588, which is associated with DBP levels [43]. Importantly, high BDP levels are associated with a longer half-life of 25OHD [43]. The second association reaching genome-wide significance in the AFR GWAS was a new association on chromosome 2. The lead SNP (rs146759773) at that locus explained ~70% (0.0035/0.0048, **Table G in S2 Appendix**) of the variance explained by the top QTL (*GC* signal shared across ancestries). This is a notable example of how the large genetic diversity in AFR populations can provide new insights despite much smaller samples (N_AFR_ = 8,306; N_EUR_ = 421,867), and highlights the importance of diverse cohorts in genomic studies, as previously suggested by others [17,18]. The lead variant (rs146759773) at that locus points to a putative new gene (*PRKD3*) involved in the regulation of 25OHD levels. Based on the Ensembl Regulatory Build, rs146759773 is predicted to overlap an enhancer region involved in gene regulation in endodermal cells and foreskin melanocytes [44]. A phenome-wide association analysis across 3,302 unique traits from the GWAS Atlas [37] showed that the top association for *PRKD3* is with heel bone mineral density in individuals of European ancestry, which could be a reflexion of abnormal 25OHD levels. However, given the low frequency of rs146759773 in European individuals, we were not able to assess the association between this locus and 25OHD in the larger European sample. It is important to note, however, that while the QTL identified is intronic to *PRKD3*, it is also near (< 500 Kb) several overlapping genes (**Fig G in S1 Appendix**), so it is possible that this association reflects the effect of another gene in the region. Using transcriptome data from sun-exposed skin to predict the expression of these genes provided evidence that four other genes in the region (but not *PRKD3*) might have expression patterns associated with 25OHD levels. However, none of the proteins encoded by the four genes has a clear link to 25OHD. *NDUFAF7* encodes a protein involved in the assembly and stabilization of Complex I, a large multi-subunit enzyme in the mitochondrial respiratory chain. *QPCT* encodes an enzyme responsible for the presence of pyroglutamyl residues in many neuroendocrine peptides. *HEATR5B* is a protein-coding gene predicted to encode a protein involved in endocytosis, protein localization, and retrograde transport of endosomes to the Golgi. *EIF2AK2* encodes a protein kinase that plays a key role in the innate immune responses against multiple DNA and RNA viruses. Although summary statistics were not available to impute the expression of *SULT6B1*, this is a candidate of interest in the region of association. It codes for a protein of the sulfotransferase family, which could be involved in sulphonation of 25OHD, affecting its half-life. A gene from the same family (*SULT2A1*) was previously implicated in a standard QTL GWAS and in a vQTL GWAS of 25OHD in EUR individuals [14]. Further analyses of this region of association are warranted. Yet, since the associated variant is predicted to overlap an enhancer region, it is possible that the association with 25OHD levels is driven by a gene at a greater distance. We note, also, that when interpreting these results, it is important to consider that the SNP allele frequency is likely to vary considerably within sub-populations from the broad group of inferred African ancestry. For example, in the 1000 Genomes Project, rs146759773 is reported to have MAF of 0% in West Gambia and 3% in the Luhya people in Webuye, Kenya [42]. This is relevant since our UKB AFR sample was defined based on genetic similarity to these 1000 Genomes Project populations. Hence it is likely to include individuals from different AFR sub-populations where the variant may be common (MAF > 1%), and others where it does not segregate (MAF = 0%).

In addition to the new findings in non-Europeans, our study adds to the literature of 25OHD in several other ways. First, we show that by combining association results from different ancestry groups, we can derive further information to fine map previously associated loci. We provide empirical evidence of how the LD structure characteristic from individuals with African ancestries (even with a small sample size) can be leveraged to prune risk variants that are in high LD regions in more recent populations. This is important given that most GWAS loci identified are in non-coding regions where the consequence of the variants is not straightforward, making it challenging (sometimes impossible) to discern from many variants in LD which one is driving the association. Indeed, cross-ancestry fine-mapping has been an active area of research for over a decade and different methods have been developed to combine information from different sets of tag SNPs across different populations to narrow down the number of candidate causal variants [45].

Second, we report for the first time three loci with dominance patterns of inheritance. These effects were identified using the same significance threshold (P < 5 x 10^−8^) used to identify additive effects, which, given the larger number of independent loci under a dominance structure, is a less stringent cut-off. Nevertheless, the associations that we observed would still be significant if we had used an over-conservative Bonferroni correction assuming that all 8.5 million variants tested were independent (*P* < 5.9 x 10^−9^, for alpha < 5%). Two of the three dominance loci identified were closely colocalised on chromosome 11 but we show evidence that they are unlikely that they tag the same causal effect. In fact, we also saw additive effects in this complex region of LD that were independent of each other despite being physically close (<50 Kb) [14]. As expected from theory [46,47], the dominance effects detected were in large QTLs (**Table K in S2 Appendix**, panel a), and the variance explained was lower than the variance explained by the additive effects at the same loci. Interestingly, the locus with largest dominance effect was led by a low-frequency variant (rs116970203:A) with opposing dominance and additive effects. While the minor allele is associated with lower 25OHD levels, a dominance effect offsets that decrease. This is consistent with Wright’s physiological theory that dominance effects protect metabolic pathways from small perturbations–with partial reductions in enzyme levels in heterozygous individuals having less disturbing effects than a homozygous deletion of an enzyme [48]. Indeed, rs116970203 is a splice QTL (in sun-exposed and non-exposed skin) and an eQTL (in skeletal muscle) for *CYP2R1* [36]–the gene that codes for the enzyme responsible for the first stage of vitamin D metabolism–and the RVIS (Residual Variation Intolerance Score) gene score for *CYP2R1* shows that only 25.09% of genes in the ExAC data are less tolerant to variation [49], suggesting that there is selection against variability/perturbations in this gene. The two other dominance loci identified were not intolerant to variation, but the dominance effects were also of much smaller magnitude in those loci (up to 23% of that seen for the top dominance signal). Importantly, the dominance effects at those loci either opposed (rs11542462:A) or supported (rs10766194:A) the additive effect both in the direction of increased 25OHD levels (**Table K in S2 Appendix**, panel a), suggesting that dominance effects play a role in protecting from vitamin D deficiency.

**Fig 4:**
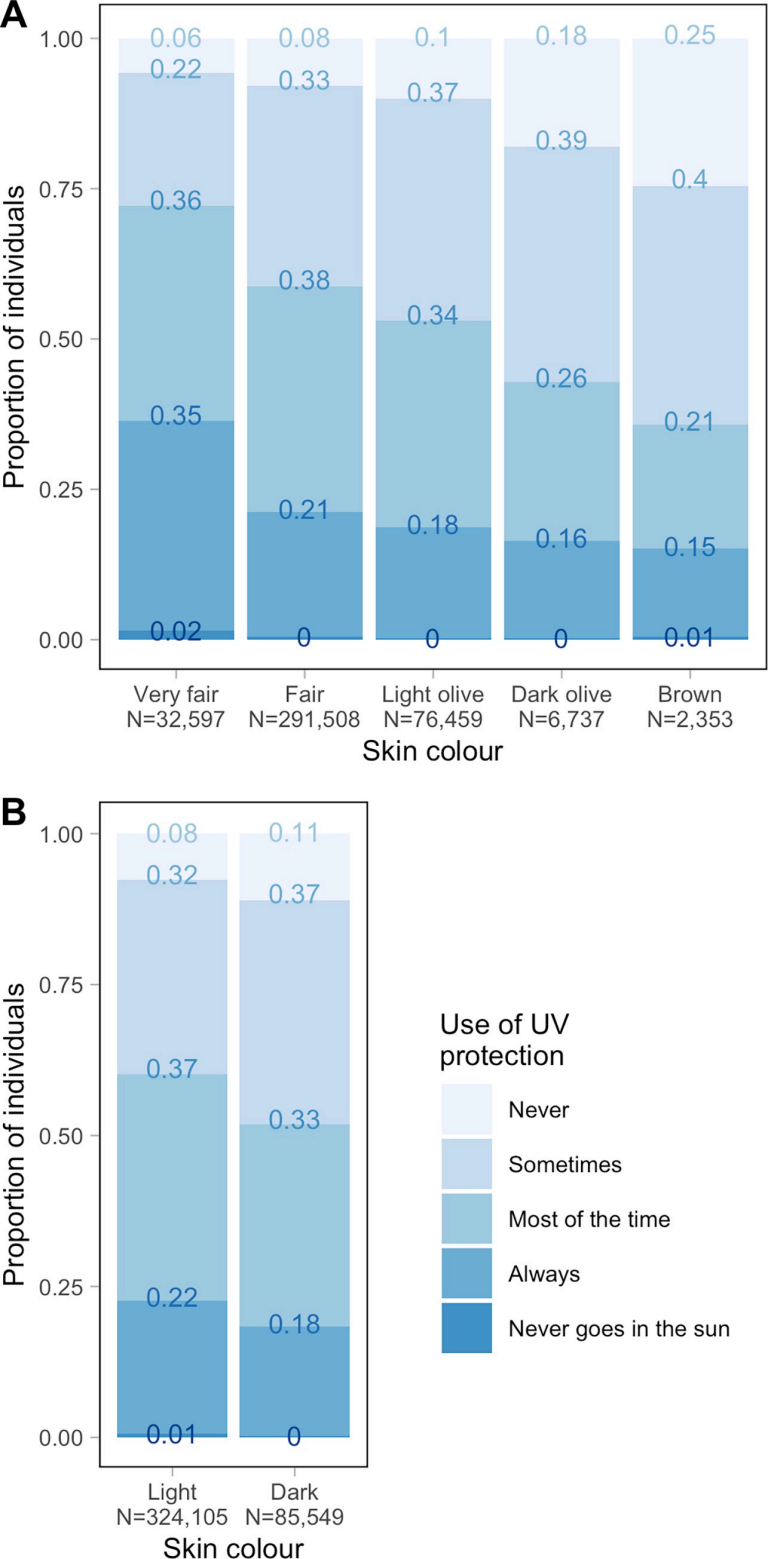
Association between skin colour and use of UV protection in EUR. Proportion of genetically-inferred EUR individuals with different skin colours and varying levels of UV protection use (y axis). Proportions are shown based on (A) skin colour reported in the UKB (data-field 1717), or (B) skin colour groups used in the skin-colour-stratified GWAS.

Third, we leveraged information from self-reported skin colour to assess if there were genetic variants associated with 25OHD through skin pigmentation pathways. Our approach to stratify the 25OHD GWAS by skin colour uncovered new GWS associations with 25OHD that were enriched for associations with traits such as skin colour and ease of skin tanning. This could seem counterintuitive, as we remove variability in skin colour from the stratified analysis. Yet, we still allowed for inter-individual variation in skin colour in the groups that we analysed (grouping two or more self-reported skin colours into the same category). Importantly, evidence from previous studies [22,50] supports the associations that we observed with this approach (see below), suggesting that real signal was not taken out through PC covariates when stratifying the analyses by two broad skin colour groups. The top two associations (rs16891982 and rs12913832) identified in the stratified GWAS implicated two well-known pigmentation genes, namely *SLC45A2* and *HERC2*, suggesting a pleiotropic effect of these genes in skin colour and 25OHD. These findings are in line with previous reports of association between these loci and 25OHD in individuals with African ancestry [22,50]. Specifically, variants in the *SLC45A2* and *HERC2-OCA2* loci, together with variants in *SLC24A5*, were shown to explain a large proportion of variance in skin pigmentation and to associate with vitamin D deficiency in a sample of nearly 600 African Americans [22]. In addition, rs16891982 was shown to associate both with skin melanin index and 25OHD levels in an independent sample of 50 self-reported African Americans and 50 self-reported European Americans [50]. Importantly, the association between rs16891982 and 25OHD levels in the second study [50] was no longer significant when adjusting for skin colour and/or ethnicity, suggesting that pigmentation and/or genetic background underlies the association. Asides from these two loci with previous links with pigmentation, our analysis uncovered a third locus on chromosome 16 that was only weakly associated (P < 10^−3^) with 25OHD in previous GWAS. The lead variant (rs35749740) is in a complex region with several overlapping genes, and it is associated with the expression of *URAHP*, *CDK10*, and *RP11-104N10*.*2* in skin and liver [36]. While the mechanism (or the exact gene) through which this variant associates with 25OHD is unclear, it is notable that the variant was also found to associate with pigmentation traits across different GWAS [37]. When interpreting these results, it is important to note that this analysis was restricted to UKB participants of inferred EUR ancestry, who showed complex relationships between skin colour and behaviours related to UV exposure in association with 25OHD levels (e.g., see **Fig 4** showing the association between skin colour and use of UV protection).

Lastly, we identify two loci with different effects across individuals with different skin colour. The first (rs10832254) is located on chromosome 11 and is an eQTL for both *RRAS2* and *COPB1* in sun-exposed skin [36]. The second (rs1352846) is in perfect LD (r^2^ = 1) with one of the key missense variant in *GC* (rs4588). In both cases, the minor allele (rs10832254:G and rs1352846:G) attenuated the difference in 25OHD levels between individuals with very fair and fair and/or light olive skin. This is relevant since there is a widely known association between skin colour and vitamin D status, which can influence decisions on uptake of vitamin supplements. In particular, the genotype at rs10832254 seems relevant when addressing bone health. The reference allele at that locus (rs10832254:A) is associated with higher 25OHD levels [14] and with increased expression of *RRAS2* [36], which in turn is up-regulated in osteoblasts and in their differentiation [51,52]. Conversely, carriers of the minor allele (rs10832254:G) tend to have lower vitamin D levels and lower expression of the osteoblast-specific gene. Our GEI results suggest that the latter, who are more likely to need vitamin D supplementation, are also more likely to have similar supplement needs regardless of skin colour differences. Further studies are needed to test this hypothesis. We note that our approach to exclusively test vQTLs for interaction with skin colour took advantage of the fact that GEI can be inferred from variants associated with variance of a trait [38], by-passing the need to test variants across the whole genome, which would incur in a high multiple testing burden. Indeed, when we conducted GWAS with interaction terms for the two skin colour groups that showed a significant interaction with vQTLs, we found no GWS associations. Yet, one of the interactions that we identified with a vQTL was in moderate LD (r^2^ = 0.4) with one of only four associations with suggestive (*P* < 1 x 10^−6^) evidence in the GWAS.

Low vitamin D levels are associated with a broad range of health outcomes, from the well-known bone health problems (e.g., rickets in children and osteoporosis in adults), to chronic diseases like multiple sclerosis and cardiovascular disease [6]. This is reflected in a large number of clinical trials using vitamin D as an intervention (1,239 listed in https://clinicaltrials.gov/ as of November 2022). Yet, protocols generally do not account for ancestry differences, and while skin pigmentation is a widely recognised risk factor for low vitamin D, the same guidelines are typically used to assess vitamin D “deficiency” across individuals [53–56]. Despite the ongoing debate on the threshold used to define vitamin D deficiency, the cut-offs discussed, and widely used in research, are based on samples that are predominantly of European ancestry [57]. One aspect that is often overlooked is that that although ancestry groups with dark skin have higher rates of vitamin D deficiency (according to definitions based in European-centric samples), the low 25OHD serum levels in those groups is not associated with significant rises in the rates of bone disease–the main outcomes justifying identification of those vitamin D deficient [58,59]. Thus, it is likely that vitamin D is an example biomarker whose calibration is not generalisable across ancestry groups. The ancestral context in which vitamin D is evaluated is clearly important, and our study further emphasizes the need to consider the genetic background and/or skin colour when assessing vitamin D status and its implications. Further research is needed to establish the validity of currently-used guidelines across different groups.

In sum, we show that 25OHD is a trait with tight links to skin colour, and by leveraging information from skin pigmentation, both within and across ancestry groups, we identify new putative genes associated with the trait, providing new insights into the biology of vitamin D. Studies across individuals of different ethnic backgrounds provide contradictory evidence as to whether it is skin pigmentation or ethnicity that contribute to variation in vitamin D levels [60–62]. Our skin-colour-stratified GWAS within a single population of European individuals supports the hypothesis that skin colour plays a role, at least partly, in determining 25OHD levels independently of ancestry background. Lastly, we provide evidence of genetic effects that vary with skin tone, providing further insight into the complex relationship between 25OHD and skin pigmentation.

## Methods

### UK Biobank sample and phenotypes

The UK Biobank (UKB) is a prospective population-based cohort of over 500,000 individuals recruited through 22 centres across the UK between 2006 and 2010 [63]. Participants (aged 40–69 years old) were comprehensively phenotyped, answering broad health and lifestyle questionnaires. Participants also provided biological samples (blood, urine, and saliva) and access to health-related records. The UKB study was approved by the North West Multi-Centre Research Ethics Committee. Informed written consent was obtained by the UKB team from each participant with opt-out allowed at any time. Data used in the current study were accessed through project 12505 in May 2023.

UKB participants were genotyped using two genotyping arrays (Affymetrix UK BiLEVE Axiom Array and UK Biobank Axiom Array). Data were quality controlled and imputed to the Haplotype Reference Consortium (HRC) [64] and UK10K [65] reference panels by the UKB team as described in Bycroft et al. [66]. UKB participants were assigned to broad ancestry groups using the same two-step approach described previously [28]. First, genetic principal components (PCs) were computed for 2,000 participants of the 1000 genomes project (1KGP) [29] that were of European (EUR), South-Asian (SAS), East-Asian (EAS) or African (AFR) ancestry. Then, UKB participants were projected onto the 1KGP PCs and assigned to the closest ancestry based on the first three PCs (**Fig S in S1 Appendix**). Distance was defined as the posterior probability, under a multivariate Gaussian distribution (over the number of PCs used to call ancestry), of each participant belonging to one of the four superpopulations. Using this method, which generalizes the k-means method and takes into account the orientation of the reference cluster to improve the clustering, we inferred the ancestry as 462,752 EUR, 11,804 SAS, 2,481 EAS and 9,159 AFR. **Note F in S1 Appendix** discusses the approach used for ancestry assignment in more detail.

Since both the HRC and the UK10K are dominated by European samples, the non-European UKB samples were re-imputed to the ancestrally-diverse 1KGP reference panel, as described in Wang et al. [28]. Briefly, within each ancestry group, individuals with genotype call rates > 0.9 were selected and variants with Hardy–Weinberg equilibrium (HWE) *P*-value > 0.001 and missing rate < 0.05 were phased using SHAPEIT2 (v2.r790) [67] and used to impute data to 1KGP using IMPUTE2 (v2.2.2) [68]. SHAPEIT2 and IMPUTE2 were both run with the default settings, using the 1KGP as reference (reference dataset downloaded from the IMPUTE2 website–March 2012 release). The genetic map used for phasing (input to SHAPEIT2—input-map) was obtained from the same 1KGP reference dataset. Quality control criteria were applied within each ancestry group, such that variants had quality scores > 0.30, HWE *P*-value > 10^−6^, and missing genotype call rates < 0.05. In total, 70,619,723 autosomal variants were available for analyses (48,836,723 in SAS, 27,498,210 in EAS, and 50,357,912 in AFR).

25OHD levels (in nmol/L) were measured using the Diasorin Liason immunoassay in blood samples collected at an initial assessment visit (2006–2010), and/or a follow-up visit (2012–2013). Samples with 25OHD concentrations outside the range of the assay (10–375 nmol/L) were excluded from analyses. In analyses of heritability and genome-wide associations, 25OHD levels were normalised with a rank-based inverse normal transformation (RINT).

### Heritability and SNP-based heritability

Heritability and SNP-based heritability of RINT 25OHD levels were estimated within ancestry groups using a method proposed by Zaitlen et al. [69] and implemented in GCTA [70,71] (with--make-bK 0.05). The method estimates pedigree-based and SNP-based heritability simultaneously in a single model without having to remove related individuals. Pedigree-based heritability estimates are based on individuals with a genomic relationship matrix (GRM) value (approximately equivalent to coefficient of relationship [π]) greater than 0.05 (as set with--make-bK 0.05), which include proxy-pedigree relationships ranging from close-related individuals (e.g., twins, with π ~ 1, or first-degree relatives like parent-offspring, with π ~ 0.5) to more distantly related individuals (e.g., four-degree relatives like great-great-grandparents/kids, with π ~ 0.0625). SNP-based heritability estimates are based on individuals with π < 0.05 (i.e., unrelated individuals). The GRM for GREML was generated for each ancestry group using common (MAF > 0.01) HapMap3 [72] single nucleotide polymorphisms (SNPs) that were independent (r^2^ < 0.9) in 1 Kb windows (PLINK--indep-pairwise 1000 100 0.9) and had HWE *P*-value > 1 x 10^−6^, and genotype missingness < 0.1. Covariates included in the model were age, sex, month of assessment, supplement intake, and the first 10 within-ancestry PCs. Supplement intake was defined as before (based on self-reported use of supplements that include vitamins) [14], and is summarised in **Table AA in S2 Appendix**. Within-ancestry PCs (wPCs) were computed with UKB genotype calls, which were LD pruned (LD r^2^ < 0.01) and had long-range LD regions removed by the UKB team. A total of 928,439, 708,492, and 763,763 variants with MAF > 0.01, HWE *P*-value < 10^−6^ and genotype missingness < 0.05 in unrelated SAS, AFR, and EAS individuals, respectively, were used to calculate 20 wPCs with PLINK [73] (--pca approx 20). wPCs from unrelated individuals were then projected onto the complete set of individuals in each ancestry group, using GCTA [71]. Genetic correlations are not reported because the standard errors of the heritability estimates were too large (see Results).

### Genome-wide association studies (GWAS)

We conducted within-ancestry GWAS of 25OHD in the three non-European populations (AFR, SAS, EAS), as described elsewhere [14]. Briefly, 25OHD levels were normalised with a RINT and tested for association with moderately rare (within-ancestry minor allele frequency [MAF] > 0.002) genetic variants. The MAF threshold used for variant inclusion was selected to ensure that at least 10 variants were observed (minor allele count [MAC] calculated as MAC = MAF x 2N) in the smallest sample analysed (i.e. the EAS ancestry). Associations were tested using the mixed linear model implemented in the GCTA software fastGWA [74], which uses a sparse version of the GRM (described above) to account for cryptic relatedness. The sparse GRMs were generated for each ancestry group setting any relationship values below 0.05 to zero. Covariates included in the GWAS were the same as those included in the heritability analyses (see above). Additionally, we performed sensitivity analyses that specifically corrected for covariates with evidence of association with 25OHD in individual ancestry groups, including use of sun protection and/or time spent outdoors (**Note B in S1 Appendix**).

Independent associations were identified using a conditional and joint (COJO; gcta --cojo-slct) analysis. The correlation between variants was assessed in 10-Mb windows (--cojo-wind 10000) using the LD information from the GWAS sample. Only variants that were not collinear (multiple regression R^2^ < 0.9;--cojo-collinear 0.9) were selected.

Correlation between effect size estimates (*r*_b_) from different ancestry groups was computed adjusting for sampling error in each individual group, using a method that accounts for estimation error [75]. Briefly, *r*_b_ was estimated as:

$${\hat{r}}_{b}=\frac{cov({\hat{b}}_{x},{\hat{b}}_{y})}{\sqrt{\left(var({\hat{b}}_{x})-mean({se}_{x}^{2}))\times (var({\hat{b}}_{y})-mean({se}_{y}^{2})\right)}},$$

where ${\hat{b}}_{x}$ and ${\hat{b}}_{y}$ are the effect size estimates in populations x and y, respectively, and *se*_*x*_ and *se*_y_ are the respective standard errors. The standard error of ${\hat{r}}_{b}$ was calculated using the formula below:

$$t=\frac{{\hat{b}}_{Pop1}-{\hat{b}}_{Pop2}}{\sqrt{{se}_{Pop1}^{2}+{se}_{Pop2}^{2}}},$$

where ${\hat{b}}_{Pop1}$ and ${\hat{b}}_{Pop2}$ are the effect size estimates in populations 1 and 2, and *se*_*Pop*1_ and *se*_*Pop*2_ are the respective standard errors.

### Gene expression imputation

As described in the Results, we identified a new association with 25OHD on chromosome 2 in AFR. The lead variant is intronic to *PRKD3* and is rare in the other ancestry groups analysed. To gain further insight into this association in the larger EUR group, we imputed *PRKD3* expression using data from GTEx v8 [33] obtained from dbGaP accession number phs000424.v8.p2. Specifically, we used association results between genetic variants and *PRKD3* expression in sun-exposed skin to compute a polygenic score (PGS) reflecting *PRKD3* expression in that tissue for each participant of genetically-inferred EUR ancestry. The PGS was a weighted sum of trait-increasing alleles derived with PLINK 1.9 (--score). SNP effect sizes used as weights in PGS computation were estimates obtained by applying SBayesR [76] to the gene expression summary statistics. SBayesR is a Bayesian method that improves polygenic prediction relative to other methods like the standard clumping and *P*-value thresholding. We used SBayesR’s default settings. Since the locus identified in AFR includes several other overlapping genes, all within 500 Kb, we repeated this analysis for 8 other genes in the region of association (depicted in **Fig G in S1 Appendix**; no summary statistics were available for *LOC100505876* or *SULT6B1*).

### Dominance GWAS

To identify non-additive effects on top of the additive effects, we conducted a genome-wide dominance association analysis in unrelated (π < 0.05) Europeans (N = 318,681). Common (MAF > 0.01) variants with HWE *P*-value > 10^−6^ and missing genotype call rates < 0.05 were tested. The phenotype was regressed of confounder effects and normalised with a RINT. Variables regressed were age, sex, genotyping batch, assessment centre, assessment month, use of supplements and the first 20 within-ancestry PCs. Dominance effects were assessed with PLINK2.0 [77] (--glm genotypic) in a model fitting both the additive effects (with genotypes coded as 0, 1, 2) and dominance effects (with genotypes coded as 0, 1, 0). Significance of dominance effects was assessed using a $\chi_{1}^{2}$ test and the genome-wide significance threshold of 5 x 10^−8^. Even though the expected estimates of dominance effects are unbiased, this model is not orthogonal such that $E[\hat{\beta }]\neq \beta$ (see [46,78]). Therefore, we disregarded estimates of additive effects from the dominance GWAS.

As described in the Results, we identified four variants with GWS dominance effects–all on chromosome 11, less than 50 Kb away from each other. To determine if these reflected independent effects, we conducted a two-step conditional analysis. First, we regressed the phenotype (same used in the dominance GWAS, i.e., 25OHD levels regressed of confounder effects and normalised with a RINT) of both the additive and dominance effect of one of the four SNPs. These RINT residuals represent the phenotype “conditioned on” a SNP. Second, we tested the effect of each of the four SNP (fitted individually as predictor) to assess if their effect was independent from the effect of the SNP conditioned on. This two-step approach was repeated four times, each time conditioning on one of the four different SNPs.

### Stratified GWAS

Genetic variants associated with skin colour have previously been shown to also associate with 25OHD levels [22]. These variants are less likely to be identified in GWAS that use PCs to control for population stratification. To address this, we conducted skin-colour-stratified GWAS in individuals of EUR ancestry–the group with largest representation in the UKB. Specifically, EUR were split into two cohorts based on self-reported skin colour. First, we selected individuals with a consistent reply to the self-reported skin colour question (UKB data-field 1717), i.e., individuals with different answers to this question across different visits were excluded from analyses. Second, individuals who reported “very fair” and “fair” skin were classed as having “light skin colour”, and those that reported “light olive”, “dark olive”, or “brown” were classed as having “dark skin colour”. EUR individuals reporting “black” skin colour (N = 42) were excluded from the analysis. See **Note G in S1 Appendix** for considerations taken to group the self-reported skin colour groups. A GWAS was then conducted separately for the two cohorts and results were meta-analysed using a fixed-effect inverse-variance weighted meta-analysis, as implemented in PLINK [73]. The stratified GWAS were conducted as the skin-colour-agnostic GWAS described above, with PCs recomputed within each skin colour group.

### Enrichment analysis

We identified a new set of SNPs that associated with 25OHD in the skin-colour-stratified analysis but not in the skin-colour-agnostic GWAS. To assess if this set (denoted here as the test set) was enriched for associations with pigmentation traits (i.e., if this set included more SNPs associated with pigmentation traits than expected) we conducted an enrichment analysis.

First, we downloaded GWAS summary statistics for three relevant traits [37], namely skin colour, ease of skin tanning, and use of UV protection. Since some variants in the test set did not have association results available for the pigmentation traits, we restricted the enrichment analysis to the set of variants that had summary data available for all pigmentation traits, i.e., our test set was restricted to N_NEW_ variants that were new associations in the SCS GWAS and that had results available for the three pigmentation traits (numbers are detailed in **Note E in S1 Appendix**).

To define a null distribution of the number of expected associations with pigmentation traits in the test set, we defined 10,000 random sets of SNPs with N_NEW_ SNPs–the same number of SNPs as in the test set. The SNPs used to generate these random sets were sampled from the list of 103 independent autosomal loci that independently associated with 25OHD at GWS level in the skin-colour-agnostic GWAS [14] and for which there was summary data available for pigmentation traits (**Table T in S2 Appendix**). For each set, we assessed how many SNPs were associated with at least one of the three pigmentation traits at *P* < 5 x 10^−8^. This defined our null distribution.

Based on the null distribution, we then calculated the empirical *P*-value for the number of associations with a pigmentation trait seen in the test set. We used a one-tailed test to determine if the number SNPs in the test set that were associated with at least one pigmentation trait was larger than expected, i.e. the *P*-value was calculated as the number of sets in the null distribution that had at least the same number of associations with pigmentation traits as the test set, divided by 10,000.

### Power analysis

The statistical power to detect an association between a phenotype and a quantitative trait locus (QTL) is measured from the non-centrality parameter (λ) of a $\chi_{1}^{2}$ distribution [79,80] as a function of the sample size (N) and the QTL heritability ($h_{QTL}^{2}$):

$$\lambda =N\times \frac{h_{QTL}^{2}}{1-h_{QTL}^{2}}$$

where $h_{QTL}^{2}=2p(1-p)\beta^{2}$ under Hardy-Weinberg equilibrium, with *p* being the population allele frequency and *β* the effect size of the QTL.

Assuming that the estimated effect sizes and allele-frequencies from the skin-colour-agnostic GWAS were the true population values, we computed, under a range of sample sizes, the expected power (at *α* = 5×10^−8^) to detect a significant association between 25OHD levels and 132 autosomal variants that were independent (as assessed by GCTA-COJO) and genome-wide significant in the skin-colour-agnostic GWAS.

### Gene-environment interactions

Gene-environment interactions (GEIs) can result in differences in phenotypic variance across different genotype groups [38]. To determine if variants associated with the variance (vQTLs) of 25OHD represented gene-environment interactions with skin colour, we conducted a GEI analysis in 318,681 unrelated (π < 0.05) EUR. Specifically, we tested the interaction between 25 previously identified 25OHD vQTLs[14] and self-reported skin colour, using the following model:

y∼g+e+g×e,

where y is the phenotype, g is the mean-centred genotype, e is the environmental factor (i.e. skin colour), and *g*×*e* is the interaction term between the genotype and skin colour. Skin colour was coded so that very-fair-skin individuals comprised the reference group. The phenotype was pre-regressed of confounder effects as described elsewhere [14]. Briefly, (1) vitamin D levels were adjusted for selected variables (listed below), (2) outliers were removed, and (3) residuals were standardised to have mean 0 and variance 1. Each step was performed within sex (male vs. female) and supplement intake group (none, other, vitamin D or missing, as defined elsewhere [14]). Variables regressed from the phenotype were age at assessment, assessment month, assessment centre, genotyping batch and the first 40 PCs. After confounder regression, residuals across the eight groups had a mean s.d. = 18.8 nmol/L. A Bonferroni-corrected threshold (0.05 / 25 vQTLs = 0.002) was used to account for multiple testing.

To assess if significant GEI would have been identified with a genome-wide approach, we conducted GWAS with interaction terms for the skin colour groups found to interact with a vQTL, i.e. we used the same model as above but tested variants across the whole genome and restricted the environmental factor (skin colour) to the two skin colour groups that interacted with a vQTL. The GEI GWAS was conducted using PLINK1.9 [73], and the same phenotype described above. This analysis was restricted to SNPs with MAF > 0.05 as previously recommended [38].

## Supporting information

S1 Appendix Supplementary Notes and Figures. (PDF)

S2 Appendix Supplementary Tables. (XLSX)
