# Supplementary material for: Cross-ancestry analyses identify new genetic loci associated with 25-hydroxyvitamin D
Source: PLoS Genet. 2023 Nov 14;19(11):e1011033. doi: 10.1371/journal.pgen.1011033 (PMC10684098; doi:10.1371/journal.pgen.1011033)
Supplement: S1 Appendix — (PDF) [file pgen.1011033.s001.pdf]

Cross-ancestry analyses identify new genetic loci associated with 25-hydroxyvitamin D

Xiaotong Wang, Valentin Hivert, Shiane Groot, Ying Wang, Loic Yengo, John J McGrath , Kathryn E Kemper, Peter M Visscher, Naomi R Wray, Joana A Revez

Table of Contents

SUPPLEMENTARY NOTES **2**

NOTE A. ASSOCIATION BETWEEN ASSESSMENT MONTH AND 25OHD ACROSS ANCESTRY GROUPS 2
NOTE B. COVARIATE ADJUSTMENT SENSITIVITY ANALYSIS 3
NOTE C. ASSOCIATION BETWEEN SKIN COLOUR AND 25OHD LEVELS 4
NOTE D. SCS SENSITIVITY ANALYSIS 6
NOTE E. ENRICHMENT FOR ASSOCIATIONS WITH PIGMENTATION TRAITS IN SKIN-COLOUR-STRATIFIED GWAS 7
NOTE F. CONSIDERATIONS OF ANCESTRY ASSIGNMENTS 8
Genetic diversity within groups analysed 8
Comparison of genetically inferred groups and self-reported ethnicity 9
Potential impact of ancestry assignment on skin colour stratified analyses 10
NOTE G. SKIN COLOUR GROUPS FOR STRATIFIED GWAS 11

SUPPLEMENTARY FIGURES **12**

FIGURE A. 25OHD DISTRIBUTIONS 12
FIGURE B. PROPORTION OF INDIVIDUALS ASSESSED BY MONTH 13
FIGURE C. SAMPLE SIZE BY SELF-REPORTED SKIN COLOUR 14
FIGURE D. CORRELATION BETWEEN PCS COMPUTED WITHIN ANCESTRY GROUP AND 25OHD LEVELS 15
FIGURE E. MANHATTAN PLOTS OF 25OHD GWAS ACROSS THREE NON-EUROPEAN ANCESTRY GROUPS 16
FIGURE F. QQ PLOTS OF 25OHD GWAS ACROSS THREE NON-EUROPEAN ANCESTRY GROUPS 17
FIGURE G. LOCUS PLOTS FOR CHROMOSOME 2 LOCUS ASSOCIATED WITH 25OHD IN AFR 18
FIGURE H. EFFECT SIZES ESTIMATED IN EUR AND AFR FOR 114 LOCI INDEPENDENTLY ASSOCIATED WITH 25OHD IN EUR 19
FIGURE I. RS116970203 LOCUS PLOTS IN EUR AND AFR 20
FIGURE J. MANHATTAN AND QQ PLOT OF 25OHD DOMINANCE GWAS 21
FIGURE K. CONDITIONAL ANALYSES OF DOMINANCE EFFECTS ON CHROMOSOME 11 22
FIGURE L. SKIN-COLOUR-STRATIFIED 25OHD DISTRIBUTIONS 23
FIGURE M. MANHATTAN PLOT OF 25OHD SKIN-COLOUR-STRATIFIED GWAS 24
FIGURE N. QQ PLOT OF 25OHD SKIN-COLOUR-STRATIFIED GWAS 25
FIGURE O. SUMMARY OF INDEPENDENT ASSOCIATIONS IDENTIFIED IN THE SKIN-COLOUR-AGNOSTIC AND SKIN-COLOUR-STRATIFIED GWAS OF 25OHD 26
FIGURE P. POWER TO DETECT ASSOCIATIONS IDENTIFIED IN THE PUBLISHED 25OHD GWAS 27
FIGURE Q. ENRICHMENT FOR ASSOCIATIONS WITH PIGMENTATION TRAITS 28
FIGURE R. GENE-BY-ENVIRONMENT INTERACTIONS (GEI) WITH SKIN COLOUR 29
FIGURE S. ANCESTRY ASSIGNMENT PRINCIPAL COMPONENTS 30
FIGURE T. EUROPEAN PRINCIPAL COMPONENTS COLOURED BY SKIN COLOUR 31

REFERENCES **32**

SUPPLEMENTARY NOTES

Note A. Association between assessment month and 25OHD across ancestry groups

As described in the results, assessment month was the top predictor of 25OHD levels, explaining 15%, 3.6%, 4.5%, and 9.0% of the variability in EUR, SAS, AFR, and EAS, respectively. To further investigate the difference in variance explained by month across the different groups, we assessed whether the month of assessment differed greatly between ancestry groups. **Figure B in S1 Appendix** shows the proportion of individuals that were assessed in each month.

Using a multinomial logistic regression (with ancestry as a predictor and assessment month as the outcome), there were statistically significant differences in month of assessment across the different ancestry groups (**Table B in S2 Appendix**, panel b). For example, the log odds of being assessed in June vs. January increased by 0.489 in SAS as compared to EUR. Similarly, and as seen in **Figure B in S1 Appendix**, the log odds of being assessed in a month other than January was generally higher in SAS and AFR when compared to EUR, although the difference was not always significant.

Modelling the data with a linear regression showed consistent results **(Table B in S2 Appendix**, panel c). On average, EUR participants were assessed in June, and SAS and AFR participants were assessed 0.356 and 0.15 later, respectively (i.e., ~11 days and 5 days later). Overall, the month of testing differed significantly across the ancestry groups ($P = 3.6 \times 10^{-28}$), but ancestry group explained little variation in month of testing ($r^2 = 0.00029$).

An alternative, and more likely explanation is if the differences in 25OHD variance explained by month of testing reflect differences in pigmentation across the groups. Specifically, differences in sunlight exposure across the months are likely to translate into smaller differences in 25OHD levels in groups of individuals with higher pigmentation levels (e.g., SAS and AFR) as compared to those seen in groups of individuals with lighter skin (e.g., EUR), since pigmentation acts as a barrier to sunlight, reducing its effect. This hypothesis is consistent with the observed number of individuals in each ancestry group that self-reported dark or light skin colour (**Figure C in S1 Appendix**).

Note B. Covariate adjustment sensitivity analysis

In the current study, analyses were adjusted for the effect of variables with evidence of association in a model fitting all variables (**Table E in S2 Appendix**). Since there was some variation in association across ancestry groups, we determined a common set of covariates based on evidence of association across the groups, considering the trade-off between confounder correction and overfitting in the smaller non-EUR samples.

This approach could impact results when confounder variables that are specific to a given group are not accounted for (e.g., use of sun protection in SAS). To assess the impact that our covariate selection may have had in our analyses, we conducted sensitivity analyses in each of the non-EUR groups correcting only for variables that showed a significant effect ($P<0.0025$ in a model fitting all variables jointly) in the group analysed. More specifically, all analyses were adjusted for age, assessment month, supplement intake, and the first 10 within-ancestry PCs. Analyses in SAS were further adjusted for sex and use of sun protection, and analyses in EAS were further adjusted for time spent outdoors in summer. As before, we opted not to correct our analyses for BMI, given the potential for collider bias from using a heritable trait as a covariate [1].

The new covariate adjustments had little impact on the results. The correlation of effect sizes between the two types of covariate adjustment was 0.97, 1, and 0.93, for SAS, AFR, and EAS, respectively, and the addition of extra covariates did not change the independent loci reaching genome-wide significance level.

Analyses using data from participants of inferred EUR ancestry, namely: (1) skin-colour-stratified (SCS) GWAS, (2) dominance GWAs, and (3) gene-environment interactions (GEI) with skin colour, were adjusted for the same covariates used in previous GWAS of 25OHD in EUR [2–4], namely age, sex, assessment month, supplement intake, assessment centre, genotyping batch and within-ancestry PCs. Given the significant effect of time spent outdoors (both in summer and winter) and of use of sun protection, we conducted sensitivity analyses in the EUR cohort including these additional covariates. The dominance effect estimates in the analysis fitting the additional covariates was highly correlated with those estimated without the extra correction ($r = 098$). Also, the addition of extra covariates in the model did not change the SNPs showing GWS effects. In the skin-colour-stratified (SCS) analysis, the effect size estimates of the meta-analysis of light- and dark-skin GWAS with the additional covariates was highly correlated with the estimates from the analysis without extra correction ($r = 0.96$). Similarly, the effect estimates in the skin-colour-agnostic (SCA) analyses with and without correction for use of sun protection and time spent outdoors were highly correlated ($r = 0.96$). Importantly, the new loci with no suggestive evidence of association in the SCA GWAS had similar results, despite the addition of the extra covariates. Lastly, the GEI analysis with additional covariates showed a high correlation in effect estimates ($r = 0.99$), and the two SNPs (rs10832254 and rs1352846) previously identified to have GEI with skin colour still showed significant interactions at a multiple testing corrected threshold of $P < 0.002$.

In sum, further adjusting our analyses for use of sun protection and/or time spent outdoors did not change results significantly and conclusions were the same.

Note C. Association between skin colour and 25OHD levels

We conducted a number of analyses to assess the relationship between skin colour and 25OHD levels. First, we conducted a Mendelian Randomisation (MR) analysis using GSMR [5] – a generalised summary-data-based Mendelian Randomisation tool implemented in GCTA [6]. For 25OHD, we used summary statistics from our published EUR GWAS [2]. For skin colour, we used summary statistics from a GWAS by Watanabe et al. [7]. Even though both sets of summary statistics are based on data from the UKB, we have previously shown through simulation that the MR regression statistic from GSMR is not biased by sample overlap [2]. We conducted our analyses with GCTA default settings, and used a random subset of 10,000 unrelated individuals of European ancestry from the UKB as LD reference.

We observed a bi-directional effect between skin colour and 25OHD levels (**Table M in S2 Appendix**, panel a – results with no HEIDI filtering). However, when we used the HEIDI-outlier test, which excludes SNP instruments with likely horizontal pleiotropic effects on both the exposure and the outcome, the effect of skin colour on 25OHD increased (and was more significant), while the effect of 25OHD on skin colour became smaller (and less significant). Intriguingly, the effect was in the opposite direction of expected, with higher levels of pigmentation leading to increased 25OHD levels (**Table M in S2 Appendix**, panel a). To assess if behaviours related to sunshine exposure could have confounded the analysis, we repeated the analysis using summary statistics of 25OHD further adjusted for use of sun protection and time spent outdoors in summer (GWAS results derived in the current study study – see **Note B in S1 Appendix**). Results from this analysis showed a significant effect of skin colour on 25OHD levels, but still in a direction that is opposite to expected (**Table M in S2 Appendix**, panel b).

Further analysis of these confounders showed that their effect on the relationship between skin colour and 25OHD is potentially more complex. For example, the model below, which includes an interaction term between the use of UV protection and skin colour, can be used to assess if the effect of UV protection varies with skin colour:

$$\mathbf{y} = \mu\mathbf{1} + \beta_{UV}\mathbf{x}_{UV} + \beta_{SC}\mathbf{x}_{SC} + \beta_{UV:SC}\mathbf{x}_{UV*SC} + \mathbf{e}$$

Here, $\mathbf{y}$ is the vector of phenotypes (log-transformed 25OHD levels). μ is the intercept. β_{UV} and β_{SC} are the effects of using UV protection and of skin colour, respectively. $\mathbf{x}_{UV}$ and $\mathbf{x}_{SC}$ are the vectors of use of UV protection (values ranging from 1="never use" to 5="never goes in the sun"), and skin colour (values ranging from 1="very fair" to 6="black"), respectively. β_{UV*SC} is the effect of using UV protection modified by skin colour. Using this model, we see that the use of UV protection is associated with lower 25OHD levels (b=-0.021, P=6.8x10^{-14}; **Table N in S2 Appendix**, panel a). However, in individuals with darker skin the use of UV protection is associated with increased 25OHD levels (for example, increasing skin colour by one unit, from very fair to fair, the effect of UV protection changes to b= -0.021 + 0.023=0.002; **Table N in S2 Appendix**, panel a). This could be a reflection of longer sun exposure by individuals with darker skin. Indeed, adding a further interaction term for time spent outdoors in summer shows that the negative effect of UV protection on 25OHD levels reduces with longer time spent outdoors (b=-0.04 vs b= -0.04 + 0.006= -0.034) and we see a more complex relationship between these variables (**Table N in S2 Appendix**, panel b).

One key assumption of MR is that the instrument variables used are independent from confounders. Given the complex relationship between skin colour and measures of sun exposure, we assessed if there was evidence of the MR instrumental variables (IVs) violating the MR assumption of no confounding. Specifically, we computed a polygenic score (PGS) based on the IVs used in our MR analysis and tested its association with both time spent outdoors in summer and use of UV protection. The skin colour IV PGS showed a significant association with time spent outdoors (b=0.038, *P*=0.0003) and use of UV protection (b=-0.195; *P*=0), both of which confounders of the relationship between skin colour and 25OHD.

An alternative approach to assess the relationship between skin colour and 25OHD is through PGS regression. To do this, we computed a PGS based on SNPs found to associate with skin colour in a cohort of individuals from Cape Verde – an island where individuals have large variation in skin colour due to the mix of West African and European ancestry [8]. In that study, skin colour was calculated as a modified melanin index based on reflectance spectroscopy on the upper inner arm. SNP effects were estimated with Bayes R, a Bayesian method that estimates the effects of all markers jointly as random effects and which was shown by simulations to have higher power to detect true positive associations than standard GWAS methods [9,10]. For the current analysis, we selected all BayesR SNPs reported by Lloyd-Jones et al. [8], i.e., 28 SNPs with PIP (posterior probability of affecting the trait conditional on the data) greater than 0.01. Of those, 21 SNPs with a Hardy-Weinberg equilibrium exact test P-value > 10^{-6}, genotype missingness < 0.05, and minor allele count > 5 in the UKB sample of unrelated EUR were selected to compute the PGS. The PGS was a weighted sum of effect alleles, with BayesR estimates used as weights, and was computed with Plink (--score). The skin colour PGS showed a strong positive association with self-reported skin colour (b=0.118; P=0), and a negative association with 25OHD levels (b=-0.006; P=0.0039).

Collectively, our analyses show evidence of a complex relationship between skin colour and vitamin D. It is important to note, however, that these analyses were restricted to participants of inferred EUR ancestry. Phenotypic analysis across all UKB participants shows a strong negative association between skin colour and 25OHD levels (b= -0.039, P=0), which remained when accounting for the complex interactions with measures of UV exposure (**Table N in S2 Appendix**, panel c).

Note D. SCS sensitivity analysis

To assess if the new associations identified in the skin-colour-stratified GWAS were driven by analytical differences other than stratification by skin colour, we repeated the skin-colour-agnostic (SCA) GWAS using the exact same approach used in the skin-colour-stratified (SCS) GWAS – henceforth termed the new SCA GWAS. Differences between the new SCA GWAS and the published SCA GWAS[2] included (1) the number of principal components fitted (20 in the new SCA GWAS vs. 40 in the SCA GWAS presented in Revez et al.); (2) the criteria used to define the European population; and (3) the criteria used to exclude individuals from analysis – in the SCS GWAS, we only included observations from the initial assessment visit and we excluded individuals with (a) European ancestry and self-reported black skin, and/or (b) inconsistent self-reports of skin colour across visits.

Overall, the estimates of effect size from the new SCA GWAS were highly correlated ($r = 0.96$, s.e. = 0.0001) with those reported in the published SCA GWAS, with 99.8% of the effect size estimates previously reported lying within the 95% confidence interval of the effect size estimates from the new SCA GWAS.

When comparing the results from the SCS GWAS with those from the new SCA GWAS, there were 11 new loci and 13 lost loci (**Table R in S2 Appendix**). Of the 11 new loci, five had no suggestive evidence of association ($P > 1 \times 10^{-6}$) in the new SCA GWAS. All the lost loci had suggestive evidence of association ($P < 1 \times 10^{-6}$) in the SCS analysis. Eight of the 17new loci that were identified in our primary analysis (comparing SCS GWAS to the published SCA GWAS) were identified again as new loci in this sensitivity analysis. The other nine (that were nearly GWS in the published SCA GWAS results; $5 \times 10^{-8} < P < 8 \times 10^{-6}$) became GWS in both analyses. These results show that the new 17 loci identified in our primary analysis were not driven by analytical differences such as the use of a different number of PCs or the different exclusion criteria to include participants in the analysis. However, this analysis highlights the fact that loci that were close to the GWS threshold of 5×10^{-8} in the SCA GWAS and that were identified as GWS in the SCS GWAS are likely to stem from analytical differences between the stratified and unstratified analyses.

Note E. Enrichment for associations with pigmentation traits in skin-colour-stratified GWAS

To assess if the new loci identified to associate with 25OHD in the skin-colour-stratified meta-analysis were enriched for variants associated with pigmentation, we conducted an enrichment analysis of association with three traits, namely (1) skin colour, (2) ease of skin tanning, and (3) use of UV protection[7]. First, we defined the number of new loci that had GWAS summary statistics available for the selected pigmentation traits. Of the 17 new loci, 13 had associations results available for all three traits. Second, we defined a null distribution of the expected number of variants in a set of 13 SNPs that had at least one GWS association with a pigmentation trait. In total, 10,000 random sets of 13 SNPs were used to define the null distribution. These were drawn from the list of 103 autosomal SNPs that (1) independently (as assessed by conditional and joint analysis implemented in GCTA-COJO[11]) associated with 25OHD at GWS level in EUR[2], and (2) had associations results available for all three pigmentation traits (**Table T in S2 Appendix**).

Of the 13 new loci with summary results of association with pigmentation traits, five associated at GWS level with at least one of the pigmentation traits (**Table U in S2 Appendix**). This was significantly higher than expected by chance (only 2 in 10,000 random sets of 13 25OHD-associated SNPs had ≥ 5 SNPs associated with pigmentation traits; empirical P = 0.0002; **Figure Q in S1 Appendix**).

Of the 19 lost SNPs (i.e., those that (1) independently associated with 25OHD in the skin-colour-agnostic GWAS at GWS level, (2) were not GWS in the skin-colour-stratified GWAS, and (3) had no variants within 1 Mb that associated with 25OHD at GWS in the skin-colour-stratified GWAS), 14 had associations results available for the pigmentation traits. Of those only one associated with a pigmentation trait at GWS level (**Table U in S2 Appendix**). This was not statistically different from what was expected by chance (empirical P = 0.75).

Note F. Considerations of ancestry assignments

The approach that we used to assign UKB participants to broad ancestry groups (described in the first section of the Methods) leverages information from a reference dataset (in our case the 1KGP) to identify groups of individuals that are genetically similar to each other. This is a commonly used approach. For example, the same reference sample has been used by other groups to infer genetic ancestry of UKB participants (e.g., see [12–14]).

Yet, this approach is not without limitations. First, genetic ancestry is a statistical construct based on quantitative measures of genetic resemblance between individuals. However, despite being a continuum, it is typically divided into a set number of genetic ancestry groups in genomic studies based on imposed boundaries that define those clusters of individuals. In our study, we used PCA to define four genetic ancestry groups based on continental categories. Within each of these groups there is substantial diversity which can affect downstream analyses if not accounted for. For that reason, in our downstream analyses we used principal components (PCs) that were computed within each of the genetically-inferred ancestry groups (as described in the GWAS section of the Methods). These account for population stratification and/or other sources of heterogeneity within each of the groups analysed.

Second, if the reference used is not representative of the groups analysed, some inferences may be less accurate than others. For example, in the 1KGP, the African samples are mostly from Western African countries. For that reason, individuals from other parts of Africa may not be identified as accurately. If they are sufficiently close to one of the clusters in the reference sample, they are assigned to that group, but if not, they are not included in the study. This problem increases with the level of resolution of groups being inferred. In this study, we define ancestry groups at a superpopulation/continental scale, minimising this issue, while still defining groups of individuals that share a higher degree of similarity between each other than with individuals from other groups.

Lastly, if the sample being inferred (UKB in our case) includes individuals with admixed background, there is a chance that they will be incorrectly assigned to a group that does not exactly match their genetic background. To account for this, our downstream analyses were adjusted for within-ancestry PCs (wPCs), which further control for confounding effects from admixed individuals that are assigned to the broad ancestry groups.

Genetic diversity within groups analysed

Assessing the quality of a reference sample is not straightforward. However, as discussed above, if the reference does not suitably represent the individuals being inferred, the inferred groups will suffer from a higher degree of heterogeneity. One way to measure this is to assess the degree of genetic diversity in the genetically-inferred groups using F_{ST}, a measure of population differentiation due to genetic structure. To assess this in the groups analysed in our study, we identified the most extreme individuals in each of the groups based on the wPC1. More specifically, within each ancestry group, we selected the top 1% values of wPC1 and estimated the F_{ST} between that group and all other individuals from the same ancestry group. We repeated this with different definitions of the extreme group, taking the top (or bottom) 1%, 5% and 10% and computing the F_{ST} of each of these groups

against the remaining individuals. We computed the multi-locus F_{ST} using the Weir and Cockerham [15] estimator implemented in Plink (--fst). The multi-locus F_{ST} was computed across 1,129,266, 1,222,935, 1,344,858, and 1120156 SNPs in EUR, SAS, AFR, and EAS, respectively. SNPs selected for analyses were HM3 SNPs that, in the respective ancestry group, had minor allele frequency > 0.01, Hardy-Weinberg equilibrium test *P*-value > 10^{-6}, missing genotype call rate < 0.05, missing samples < 0.1, and imputation score > 0.3.

Results are summarised in **Table Y in S2 Appendix**, panel a. On average, F_{ST} was 0.0011, 0.0085, 0.0045, and 0.0104 in EUR, SAS, AFR, and EAS, respectively. To provide context on these estimates, values of $F_{ST} \leq 0.01$ are typically seen between populations of the same continent and values of $F_{ST} \sim 0.1$ are typically seen between populations from different continents (e.g., see [14,16]). Thus, our results suggest that the limitations discussed above did not affect our ancestry assignments significantly, such that the groups analysed show a degree of genetic diversity consistent to what is expected for a continent.

For comparison, we also computed the F_{ST} across ancestry groups. As seen in **Table Y in S2 Appendix**, panel b, these are in line with F_{ST} values typically seen between populations from different continents.

Comparison of genetically inferred groups and self-reported ethnicity

As part of the online questionnaire that UKB Participants filled during the initial assessment visit, they were asked "What is your ethnic background?" (Data-Field 21000). Possible answers included:

1. White (British; Irish; or Any other white background)
2. Asian or Asian British (Indian; Pakistani; Bangladeshi; or Any other Asian background)
3. Black or Black British (Caribbean; African; or Any other Black background)
4. Chinese
5. Other ethnic group
6. Mixed (White and Black Caribbean; White and Black African; White and Asian; or Any other mixed background)
7. Do not know
8. Prefer not to answer

We compared the self-reported ethnic background to the ancestry assignments in all participants included in our study (i.e., that had 25OHD levels available). **Table Z in S2 Appendix** shows the proportion of individuals in each genetically-inferred ancestry group that self-identified their ethnicity, or not ("Do not know", "Prefer not to answer" and "N/A – no answer available"). For example, 98.9% of participants that we categorised as EUR self-reported "White" ethnic background. Overall, the ancestry assignments from our PCA are mostly consistent with self-reported ethnic background.

There are, however, three important considerations in this analysis that we would like to highlight. First, ethnicity is a social construct that groups individuals based on perceived similarities (including language, religion, beliefs, etc.) and it varies globally based on perceptions. Therefore, genetic ancestry and ethnicity are not equivalent concepts.

Second, there are several reasons why self-reported ethnicity may not be correct. For example, individuals that are not fully aware of their ancestral background, or individuals that only know their more recent ancestry may self-identify with one ethnic group despite having admixed background [17]. As an example, we assessed the concordance of self-reported ethnicity in full siblings from the UKB (as defined in Bycroft et al. [18]). Although most pairs of full siblings reported the same ethnicity (22,227 out of 22,423, 99%), there were 196 pairs with discordant responses, and the level of discordance increased at the more defined ancestry groups (e.g., British; Irish; or Any other white background, instead of just "White"), with 5% (1,172 of 22,423) of full-sib pairs reporting different ancestry. For this reason, genomic studies commonly use statistical tools such as PCA to identify groups of individuals that are genetically similar to each other or to a reference sample.

Third, and most importantly, our analyses aim to map genetic variants associated with 25OHD levels. Thus, the key goal of categorising participants into broad ancestry groups is "to conduct the study in a set of individuals that are genetically more similar, rather than to infer ancestry per se" (see NIH's guidelines: Using Population Descriptors in Genetics and Genomics Research: A New Framework for an Evolving Field [19], and Coop 2022 [20]).

Potential impact of ancestry assignment on skin colour stratified analyses

We further assessed whether the ancestry assignment method used in our study could impact our skin-colour-stratified (SCS) GWAS in the EUR group. This could happen if, for example, the individuals assigned to the dark-skin colour group included admixed African ancestry individuals that were assigned to the European ancestry group. To investigate this, we plotted the within-ancestry PCs (i.e., PCs computed in the EUR group) and coloured these by skin colour group (the groups used in our stratified analyses). As seen in, **Figure T in S1 Appendix**, EUR in the light- and dark-skin colour groups were mostly overlapped. This suggests that the dark-skin group defined in our study is genetically similar to the light-skin group, and that it did not include highly admixed individuals that would affect the analyses.

We also note that in our SCS GWAS we re-computed PCs within the two skin colour groups to account for further population structure, which would capture differences from individuals with smaller degree of admixture.

Note G. Skin colour groups for stratified GWAS

To assess if within-ancestry principal components (wPCs) removed some of the variation in skin colour that is biologically meaningful for 25OHD levels, we conducted skin-colour-stratified analyses. As part of the UK Biobank (UKB) study, participants were asked in an online questionnaire "What best describes the colour of your skin without tanning?" (UKB Data-Field 1717). Possible answers were "Very fair", "Fair", "Light olive", "Dark olive", "Brown", "Black", "Do not know", and "Prefer not to answer". For our analysis, we selected individuals of genetically-inferred European (EUR) ancestry (see Methods) with a consistent reply to this question across all visits attended. We excluded from analysis individuals who replied "Do not know", "Prefer not to answer", or "Black" (given the small sample size and likelihood of this being an inaccurate reply for this genomically-inferred population).

On average, there was no linear relationship between skin colour and 25OHD levels. As expected, individuals with brown skin colour had lower 25OHD levels than those with fair and olive skin, but that was not the case when compared with individuals of very fair skin (**Table O in S2 Appendix**). While populations with dark skin colour typically show lower levels of vitamin D[21–24], we note that there are several factors that may also influence skin colour and affect this relationship within a population from the same continent. For example, we observed a significant association between the use of sun protection and the different self-reported skin colours, with individuals with darker skin using less sun protection (**Figure 4**; P = 0).

Although our analyses were restricted to individuals with a consistent self-reported skin colour, there may still be inter-individual differences in people's perception of skin colour such that people that report very fair or fair skin may be the same skin tone. To assess if there were skin colour groups with greater genetic similarity that should be grouped in our analysis, we first estimated the SNP-based heritability ($\hat{h}^2_{SNP}$) of 25OHD in a GREML [25] analysis of unrelated (coefficient of relationship (π) < 0.05) individuals in each of the skin colour groups. Except for individuals who reported brown skin (N = 1,284), all groups had similar $\hat{h}^2_{SNP}$ estimates, which were close to the estimate in EUR of any skin colour ($\hat{h}^2_{SNP} = 0.13 \pm 0.009$; **Table AB in S2 Appendix**). We then estimated the genetic correlation between each skin colour group in a bivariate GREML analysis (gcta --reml-bivar) of unrelated (π < 0.05) individuals. The genetic correlation between pairs of skin colour groups was not significantly different from 1, except when the correlation was calculated against the brown skin colour group (**Table AC in S2 Appendix**).

Given the large similarities between different skin colour groups, we classed all individuals into one of two skin colour groups. Individuals who reported "very fair" and "fair" skin were classed as having "light skin colour". Those that reported "light olive", "dark olive", or "brown" were classed as having "dark skin colour". This maximised the sample size of the dark skin colour group, while separating individuals with light and dark complexions in a way that minimised possible differences in people's perception of skin colour.

SUPPLEMENTARY FIGURES

Figure A. 25OHD distributions

25OHD distributions (nmol/L) in individuals of genetically-inferred European (EUR; N=421,867), South Asian (SAS; N=9,983), African (AFR; N=8,306) or East Asian (EAS; N=2,279) ancestry. Corresponding statistics are presented in **Table A in S2 Appendix**.

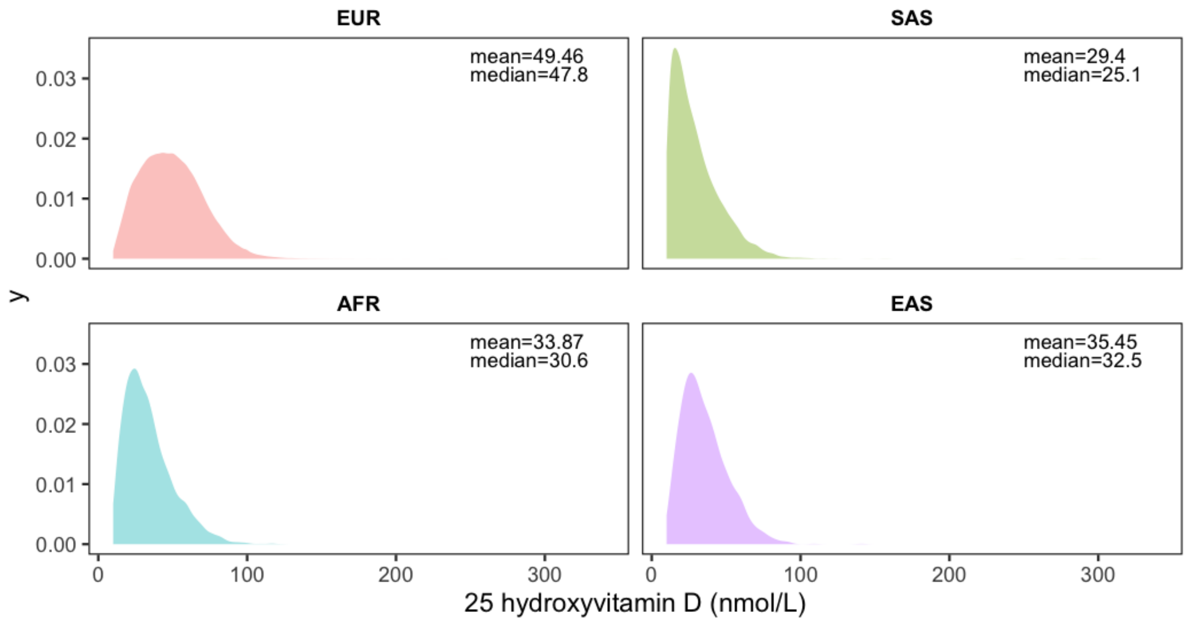

Figure B. Proportion of individuals assessed by month

Corresponding statistics are presented in **Table B in S2 Appendix**. Abbreviations: AFR, African; EAS, East Asian; EUR, European; SAS, Southeast Asian.

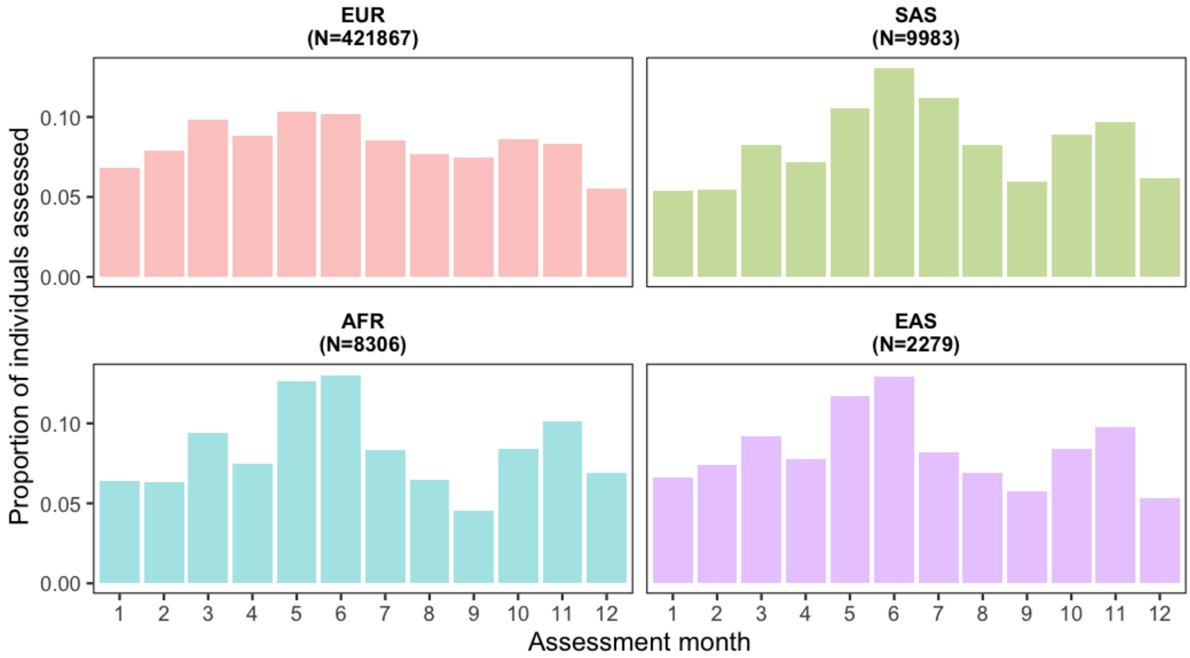

Figure C. Sample size by self-reported skin colour

Number of individuals of genetically-inferred European (EUR; N=411,826), South Asian (SAS; N=9,451), African (AFR; N=7,988) or East Asian (EAS; N=1,915) ancestry stratified by self-reported skin colour (UKB data-field 1717, initial assessment visit). Sample sizes are available in tabular form in **Table C in S2 Appendix.**

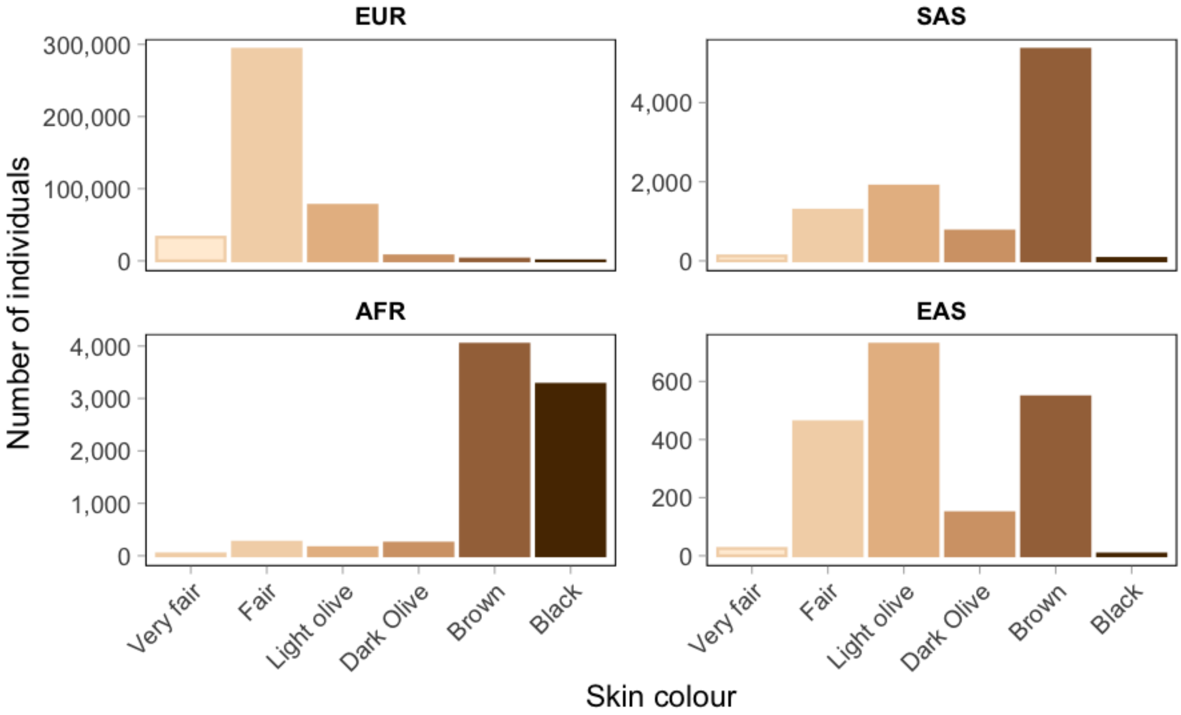

Figure D. Correlation between PCs computed within ancestry group and 25OHD levels

Association results are from a linear model fitting each within-ancestry principal component (wPC) individually. These results are also presented in **Table D in S2 Appendix**. NB: since wPCs were computed within each ancestry group, they should not be compared across ancestry groups.

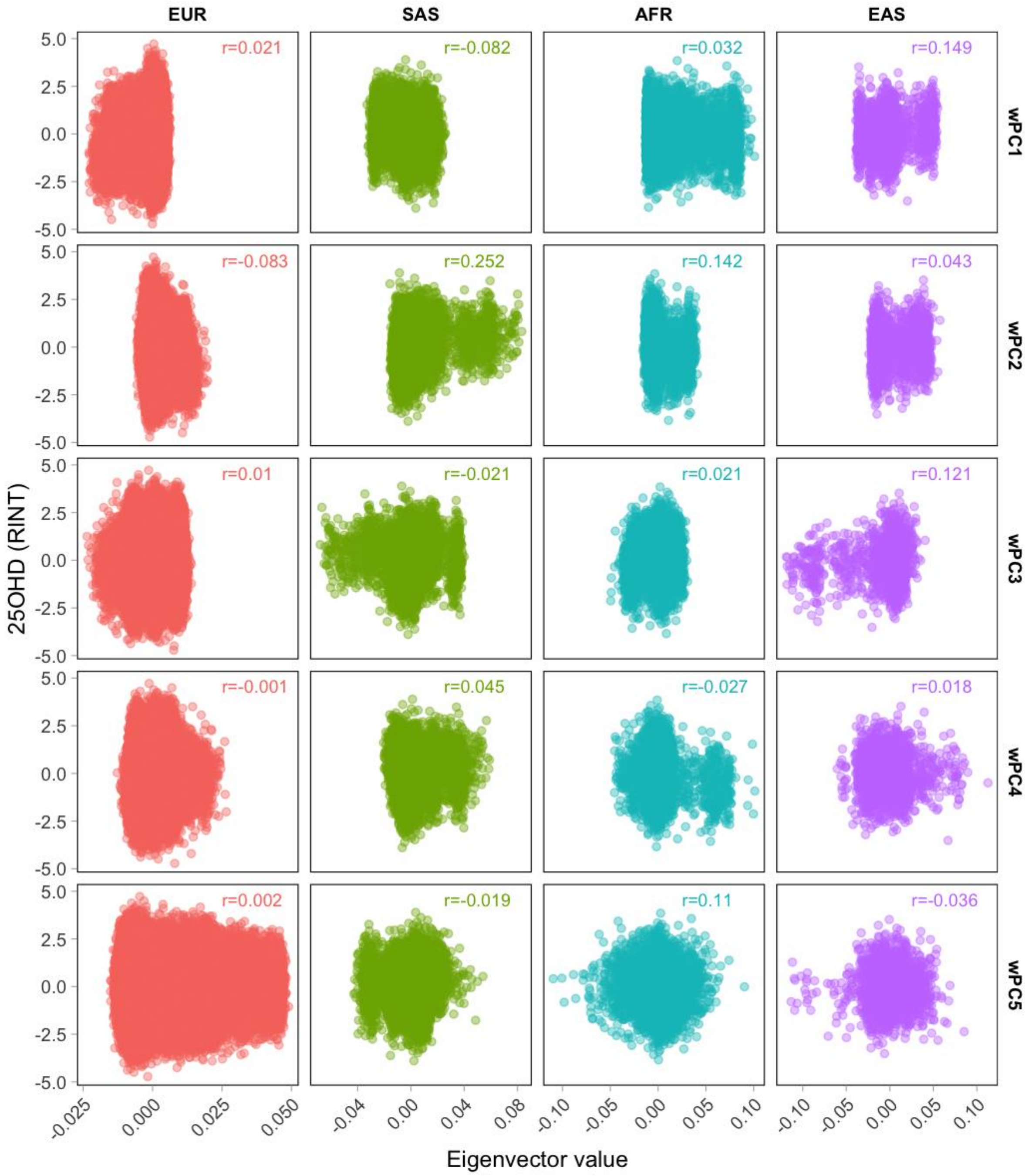

Figure E. Manhattan plots of 25OHD GWAS across three non-European ancestry groups

Manhattan plots showing the −log10 *P*-values from the fastGWA [26] association test between 25-hydroxyvitamin D (25OHD) and genome-wide SNPs in Southeast Asians (A), Africans (B), and East Asians (C) and Europeans (D). Association results for the EUR cohort were obtained from Revez et al. 2020 [2] and are shown for comparison. Red dots represent independent variants identified as genome-wide significant with conditional and joint analysis (GCTA-COJO [11]) applied to the GWAS summary statistics. The red horizontal line represents the genome-wide significance threshold of 5 x 10^{-8}.

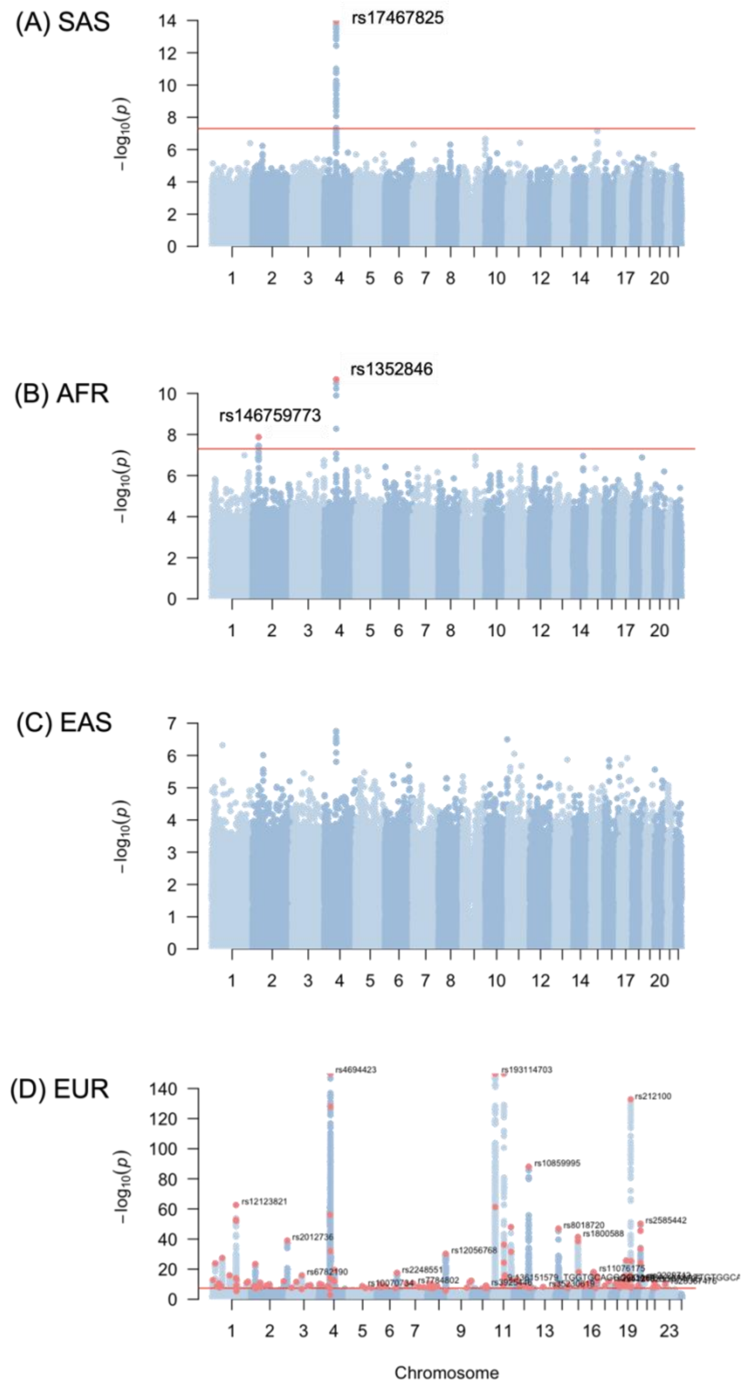

Figure F. QQ plots of 25OHD GWAS across three non-European ancestry groups

QQ plots showing the observed (y axis) and expected (x axis) −log10 *P*-values from the fastGWA[26] association test between 25-hydroxyvitamin D (25OHD) and genome-wide SNPs in Southeast Asians (A), Africans (B), East Asians (C) and Europeans (D). Association results for the EUR cohort were obtained from Revez et al. 2020[2] and are shown for comparison.

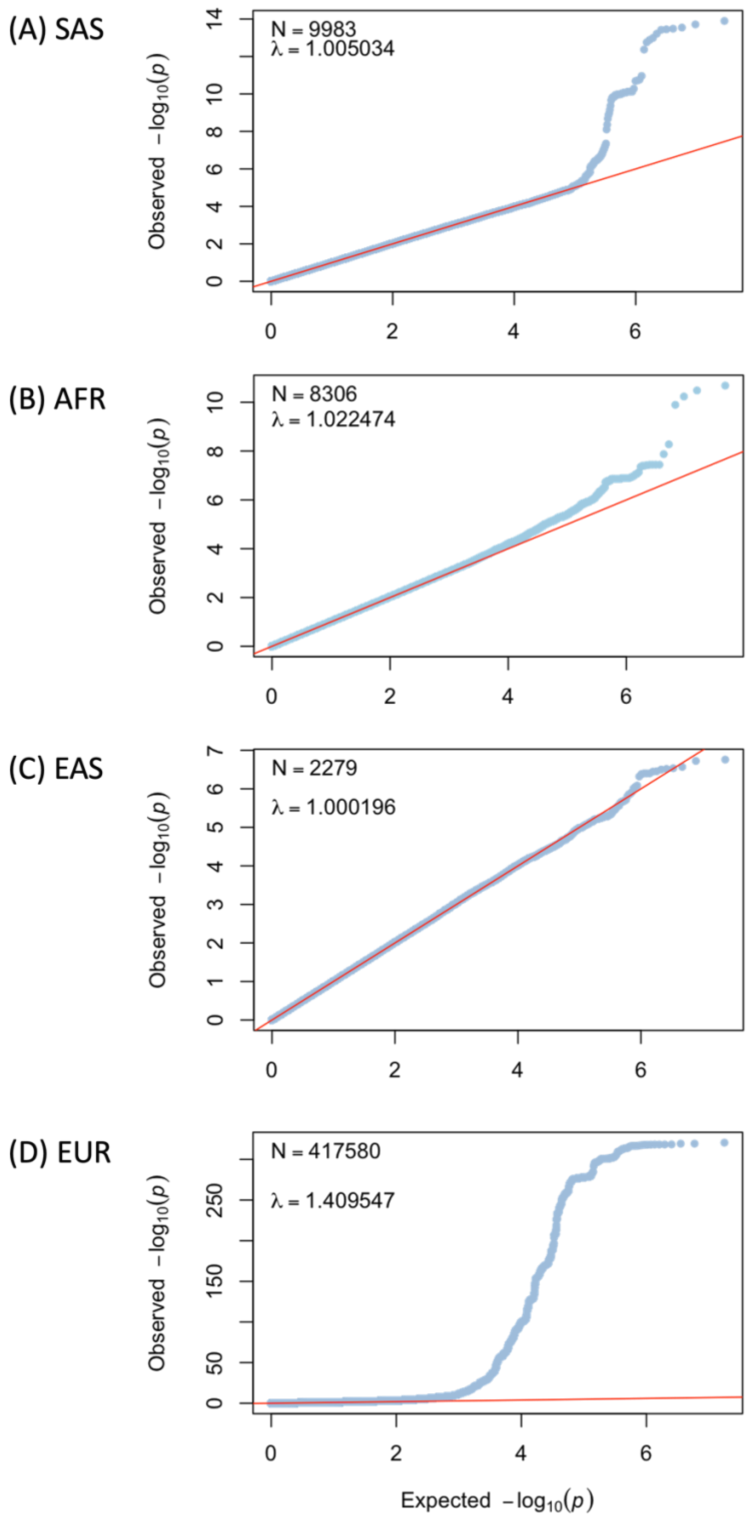

Figure G. Locus plots for chromosome 2 locus associated with 25OHD in AFR

Regional plot of associations between 25OHD and genetic variants within 500 Kb of (A) rs146759773 (purple diamond) in AFR, and (B) rs2268987 in EUR. rs2268987 is used as the index SNP in EUR since rs146759773 had low frequency (P < 0.002) and was not tested. The x axis represents the physical location of the genetic variants and the y axis the $-\log_{10} P$-value of the association with 25OHD. Association results for the EUR cohort were obtained from Revez et al. 2020[2].

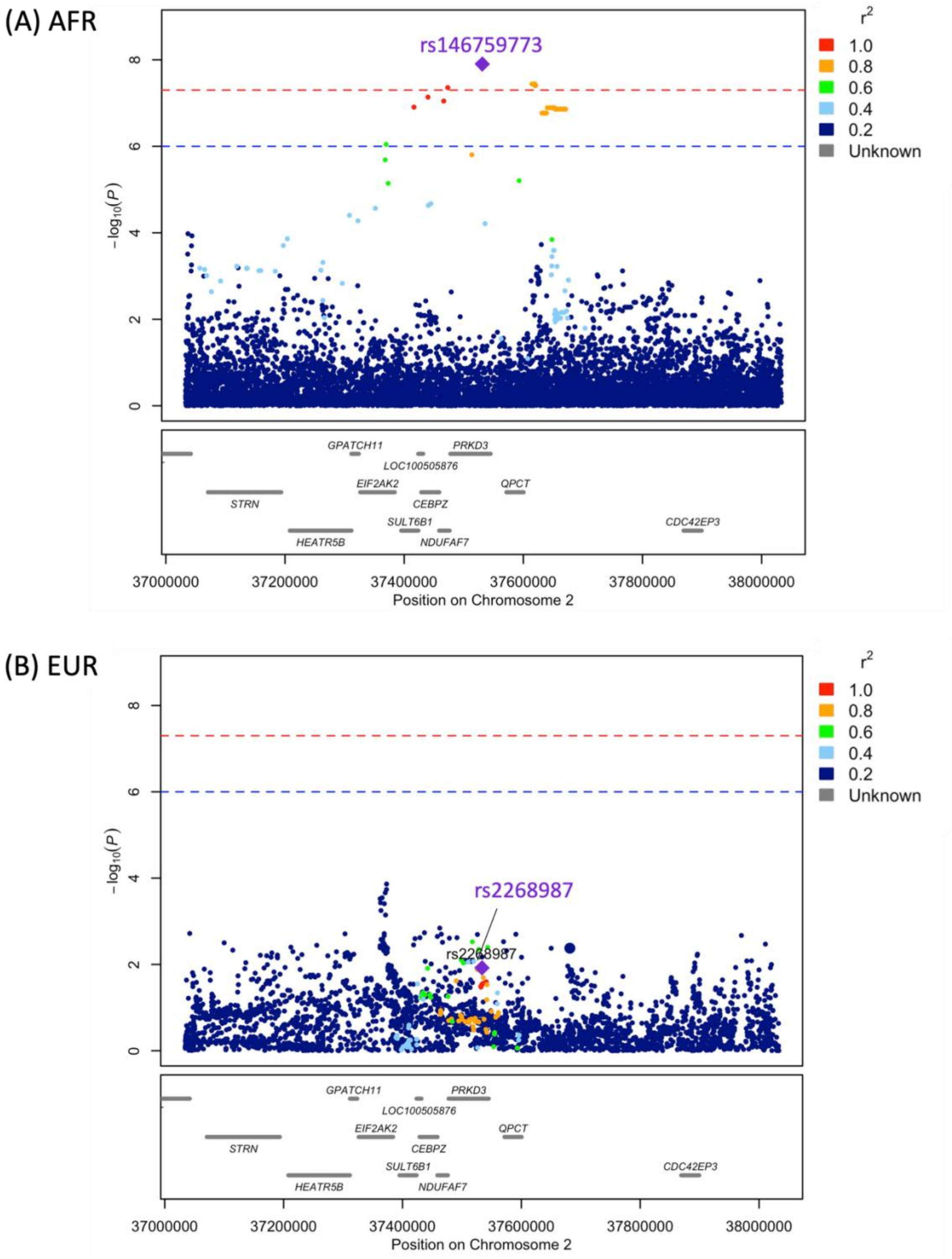

Figure H. Effect sizes estimated in EUR and AFR for 114 loci independently associated with 25OHD in EUR

(A) Marginal effect size estimates (dots) and standard errors (bars) of association with 25OHD estimated with fastGWA [26] in EUR (x axis) and AFR (y axis) for 114 variants that independently associated with 25OHD in EUR[2]. rb represents the correlation between effect size estimates in EUR and AFR (see Methods), and se_rb is the respective standard error. (B) Absolute difference in effect size estimates (y axis) and absolute difference in AF (x axis). Variants highlighted in light pink had the largest difference in effect size and smallest difference in allele frequency between EUR and AFR. Association results for the EUR cohort were obtained from Revez et al. 2020 [2]. Summary statistics are presented in **Table I in S2 Appendix**.
NB: Of the 143 SNPs identified to independently associate with 25OHD in EUR, only the 114 presented here were also tested in AFR (MAF > 0.002) and had matching alleles.
NB: The 114 SNPs presented were all independent and genome-wide significant when fitted jointly in the COJO analysis, however, these include eight variants with marginal *P* > $5x10^{-8}$.

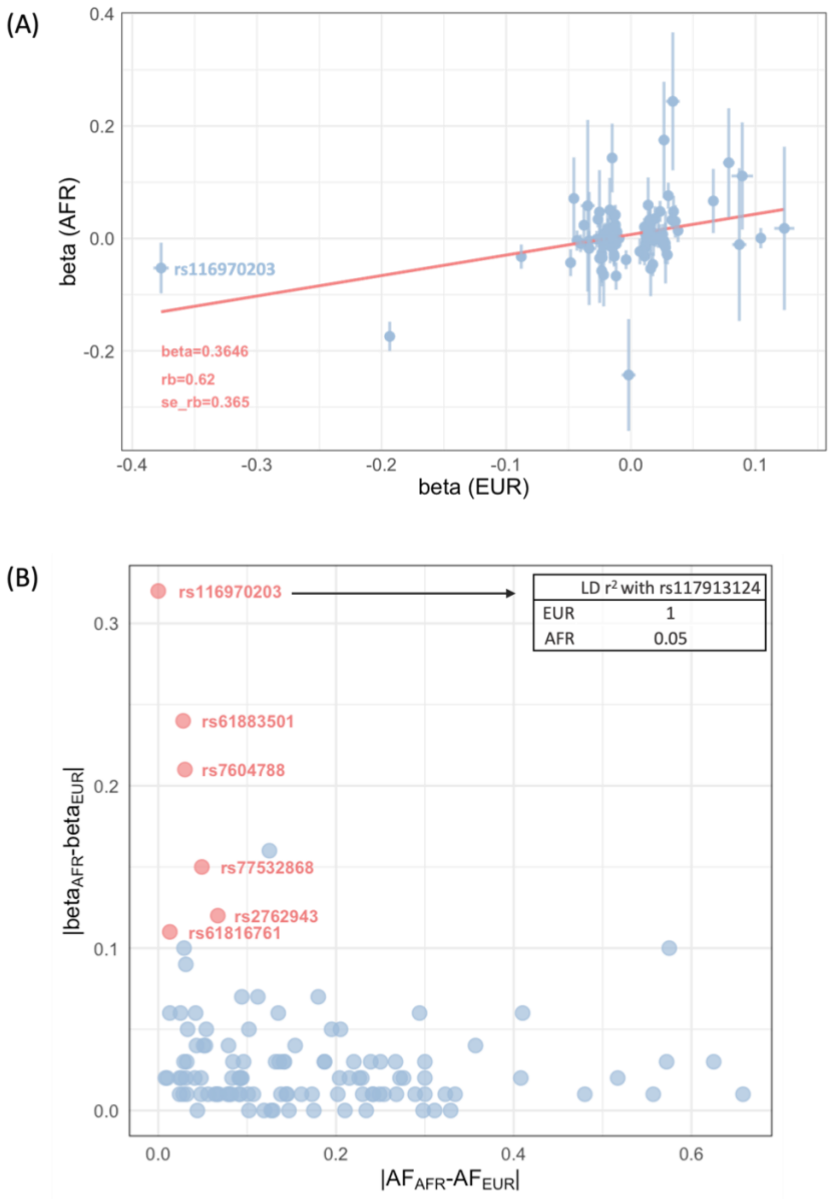

Figure I. rs116970203 locus plots in EUR and AFR

Regional plot of associations between 25OHD and genetic variants within 100 Kb of rs116970203 (purple diamond) in EUR (A) and AFR (B). The x axis represents the physical location of the genetic variants and the y axis the -$\log_{10}$ *P*-value of the association with 25OHD. rs117913124 is highlighted in both plots. Association results for the EUR cohort were obtained from Revez et al. 2020 [2].

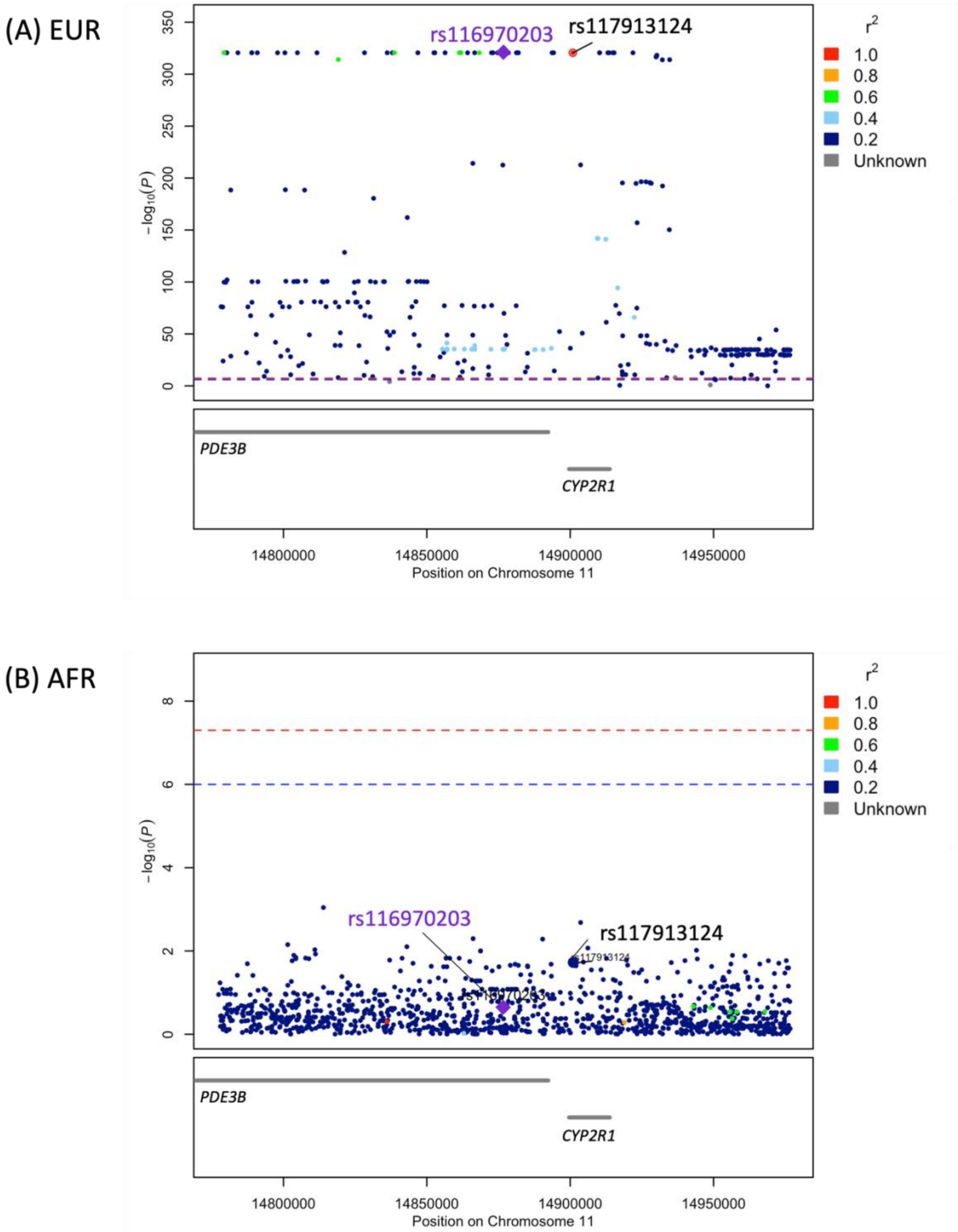

Figure J. Manhattan and QQ plot of 25OHD dominance GWAS

Association results from the dominance GWAS of 25OHD levels in individuals of European ancestry. (A) Manhattan plot showing the −log10 *P*-values from the associations. The red horizontal line represents the genome-wide significance threshold of 5 x 10^{-8}. (B) QQ plots showing the observed (y axis) and expected (x axis) −log10 *P*-values from the dominance association tests between 25OHD and genome-wide SNPs.

(A)

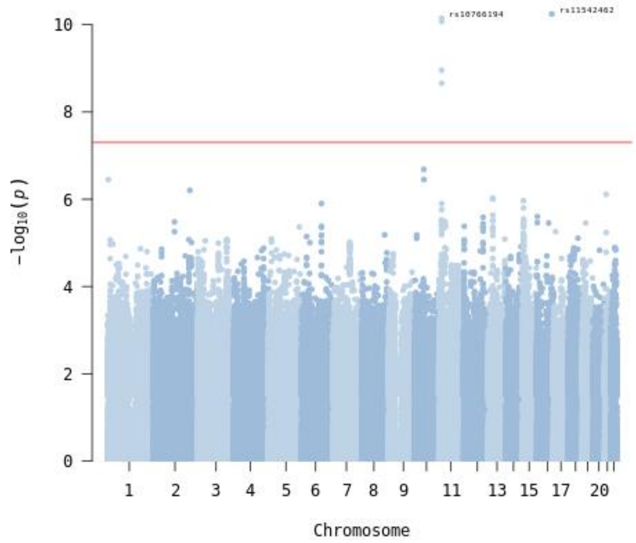

(B)

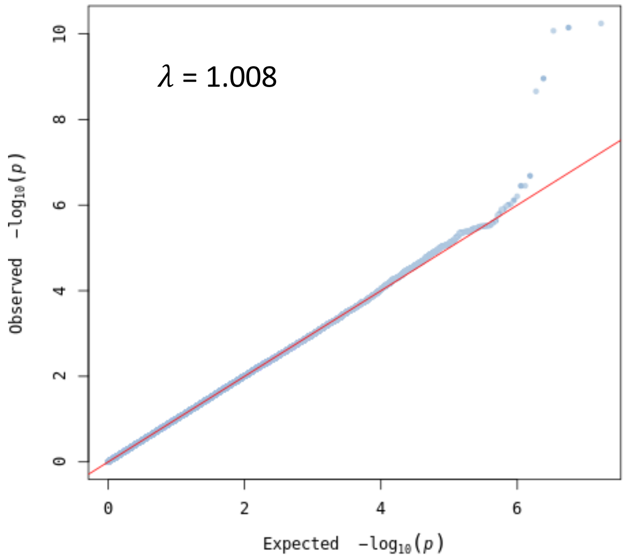

Figure K. Conditional analyses of dominance effects on chromosome 11

Dominance effects for rs1037378, rs10766194, rs116970203 and rs117913124 before and after conditioning on each of those SNPs. Results are also presented in tabular form in **Table K in S2 Appendix**.

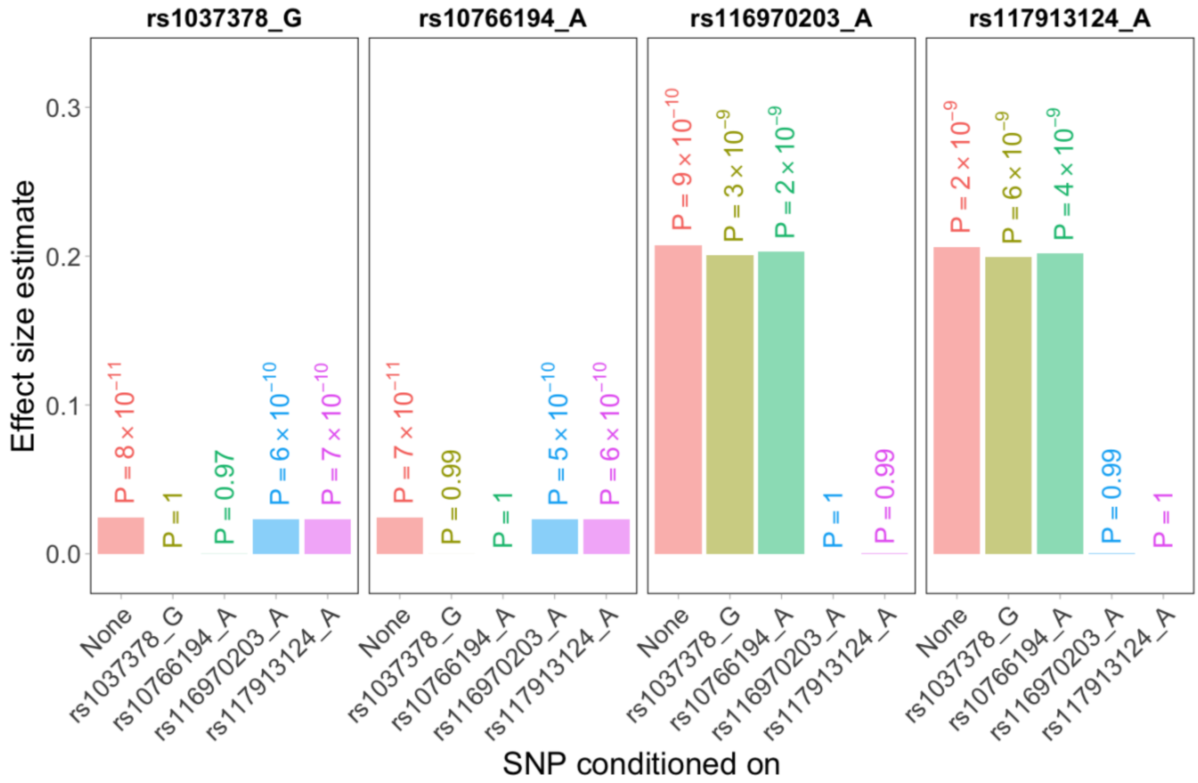

Figure L. Skin-colour-stratified 25OHD distributions

Distribution (A) and mean, variance and coefficient of variation (B) of 25OHD (nmol/L) across light- and dark-skin EUR (N_{Light}=324,105, N_{Dark}=85,549). The two skin colour groups differed significantly in mean (Wilcoxon Test P = 0) and variance (Levene's $P = 1.4 \times 10^{-30}$) between the two skin colour groups. Corresponding statistics are presented in **Table O in S2 Appendix**. The coefficient of variation was computed as the ratio of the standard deviation to the mean.

NB: Observations that were over 3 standard deviations from the mean of each stratified population were removed from analysis.

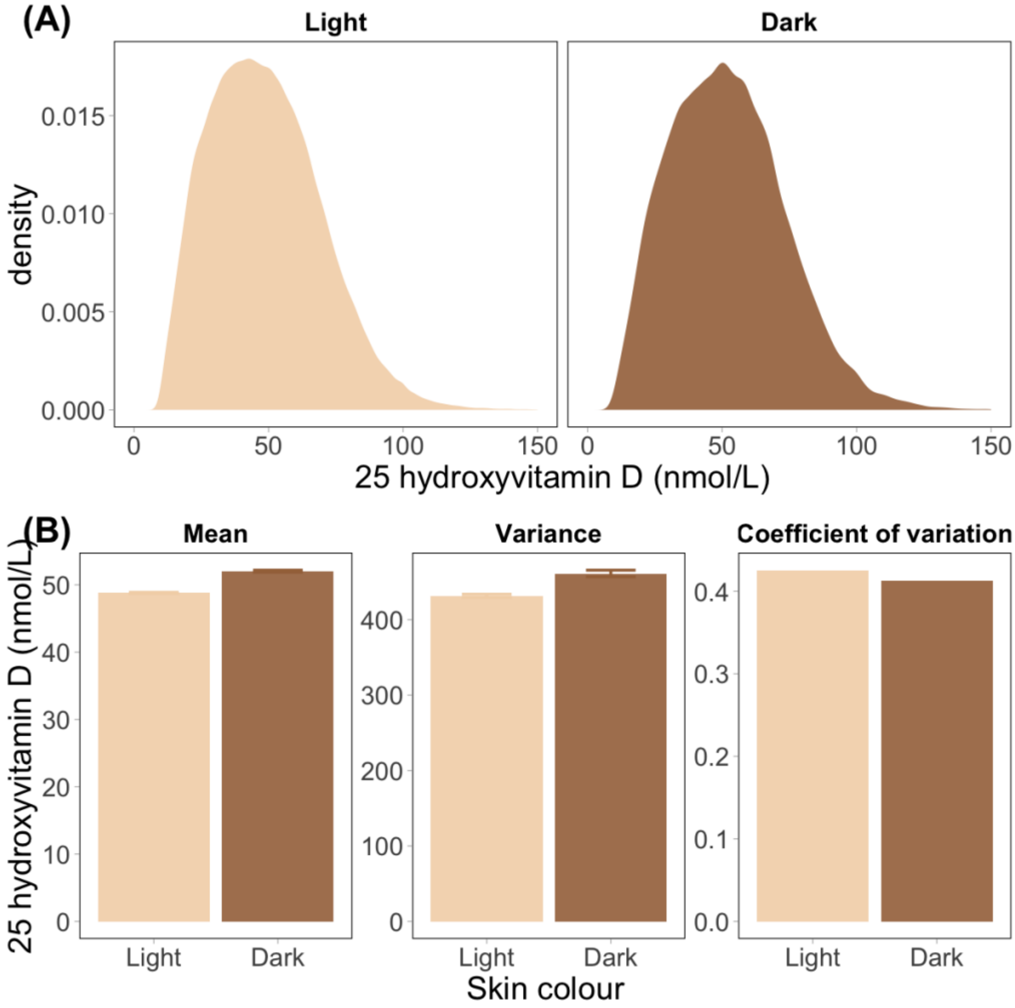

Figure M. Manhattan plot of 25OHD skin-colour-stratified GWAS

Manhattan plots showing the −log10 *P*-values in the (A) skin-colour-stratified (SCS), and (B) skin-colour-agnostic (SCA) GWAS. SCA results were obtained from Revez et al. 2020 [2] and are shown for comparison. The red horizontal line represents the genome-wide significance threshold of 5 x 10^{-8}.

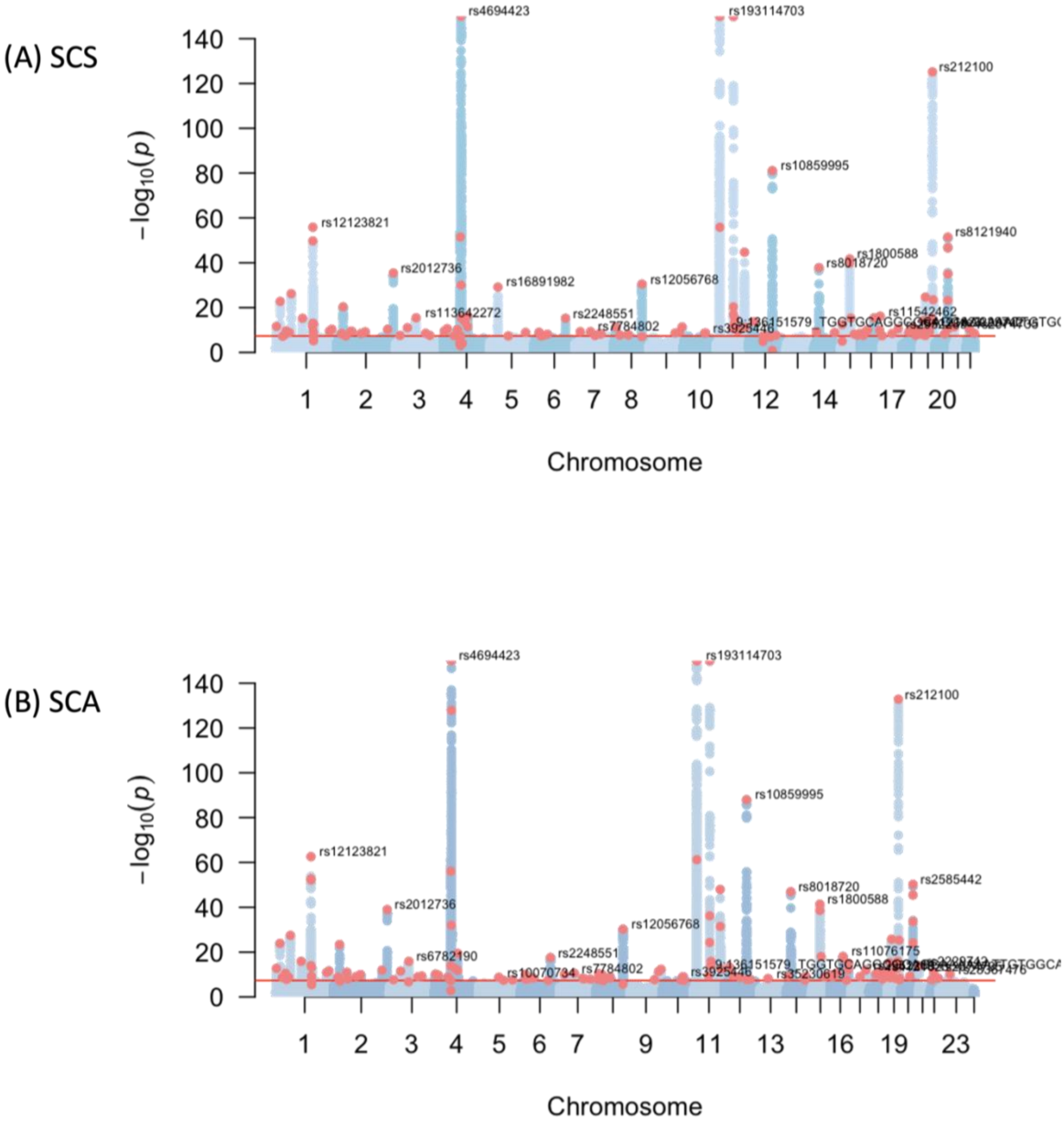

Figure N. QQ plot of 25OHD skin-colour-stratified GWAS

QQ plots showing the observed (y axis) and expected (x axis) −log10 *P*-values in the (A) skin-colour-stratified (SCS), and (B) skin-colour-agnostic (SCA) GWAS. SCA results were obtained from Revez et al. 2020 [2] and are shown for comparison.

(A) SCS

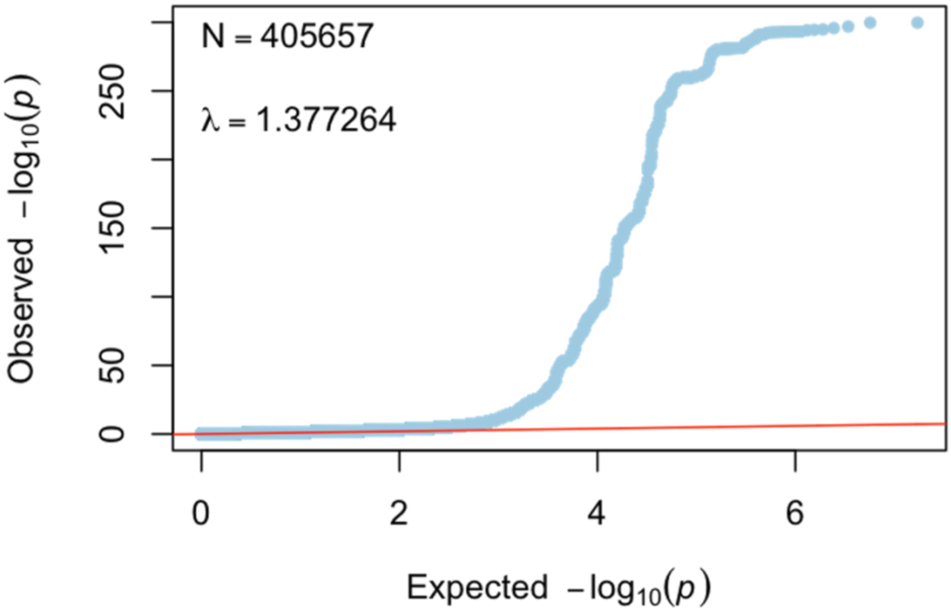

(B) SCA

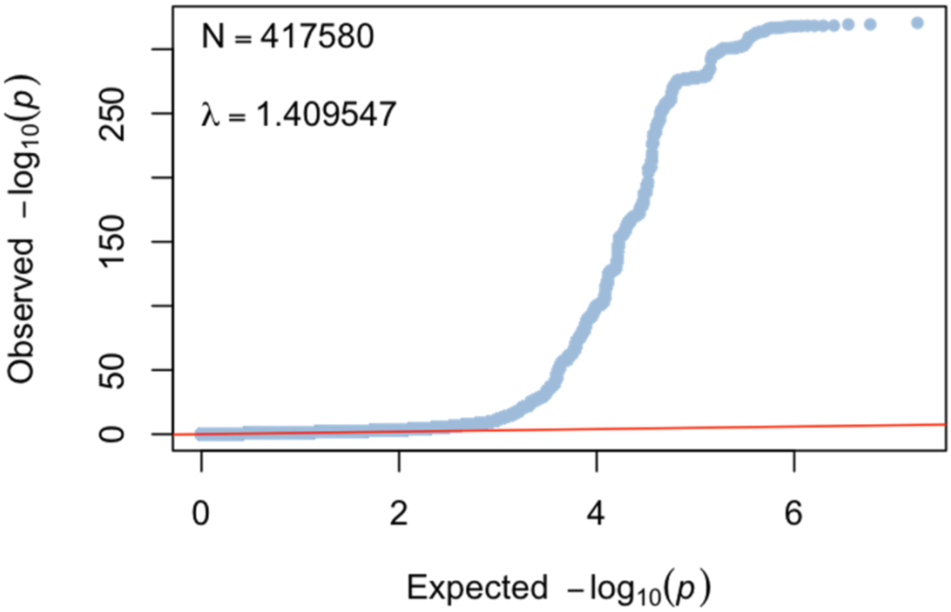

Figure O. Summary of independent associations identified in the skin-colour-agnostic and skin-colour-stratified GWAS of 25OHD

Independent associations were assessed by conditional and joint analysis implemented in GCTA-COJO[11]. Results for the skin-colour-agnostic GWAS were obtained from Revez et al. 2020[2].
NB: Only associations in autosomes were considered in the current analysis.

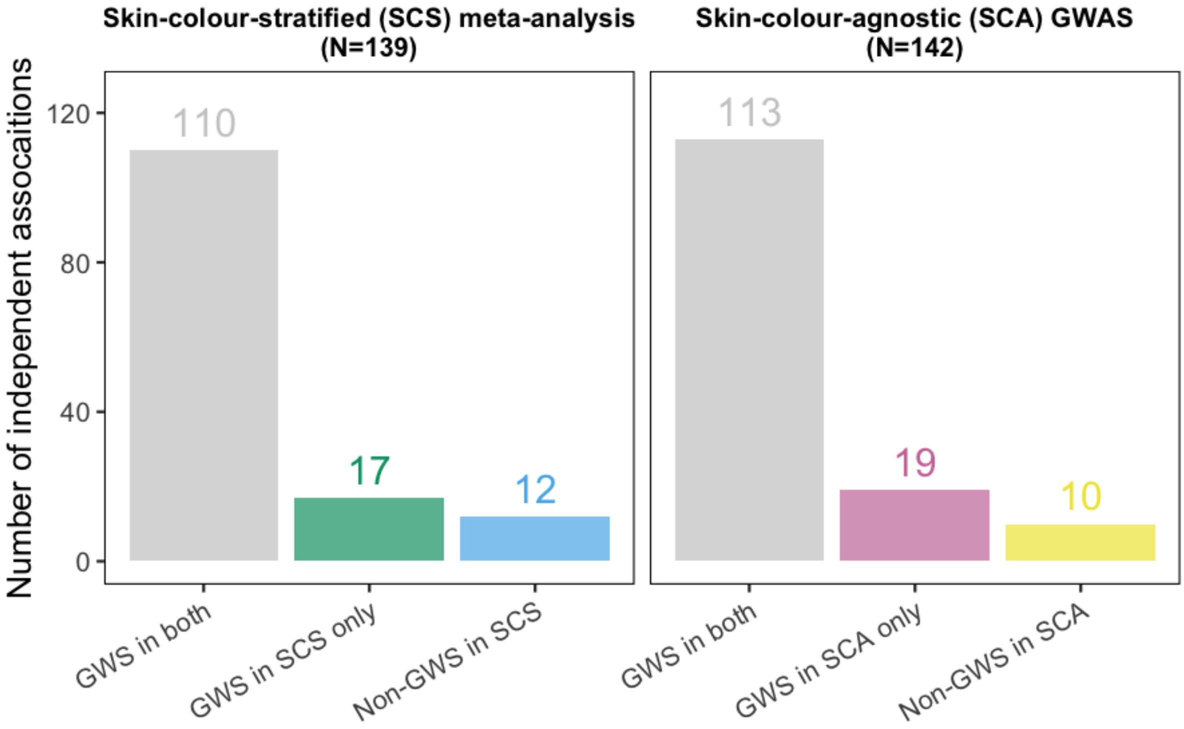

Figure P. Power to detect associations identified in the published 25OHD GWAS

Power to detect each of the 142 independent autosomal associations identified in the Revez et al. GWAS[2], assuming that the effect size estimates from that study were the true effect sizes. The horizontal dashed line represents 50% power. The vertical dashed line represents the mean sample size across all variants in the analysis. Red lines represent variants that were no longer genome-wide significant ($P > 5 \times 10^{-8}$) in the skin-colour-stratified analysis.

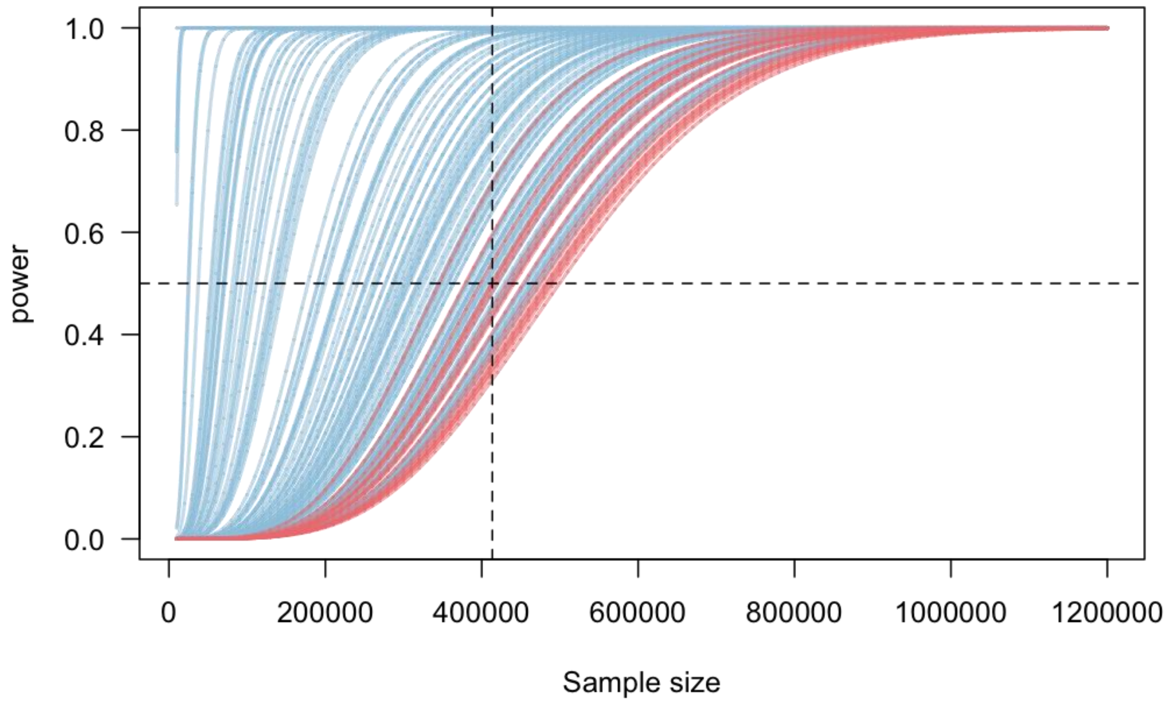

Figure Q. Enrichment for associations with pigmentation traits

The histogram in panel (A) represents the null distribution of the number of SNPs, in a set of 13 25OHD-associated SNPs, that associated with at least one pigmentation trait at GWS level (see Methods and **Note E in S1 Appendix**). The distribution is based on 10,000 random sets of 13 25OHD-associated SNPs (**Table T in S2 Appendix**). The dashed line represents the number of GWS associations with at least one pigmentation trait in the set of 13 new loci identified in the skin-colour-stratified meta-analysis (n=5; only 2 null sets have ≥ number of GWS associations with a pigmentation trait; *P* = 2/10,000 = 0.0002). The table presented in panel (B) summarises the null distribution plotted in panel (A).

(A)

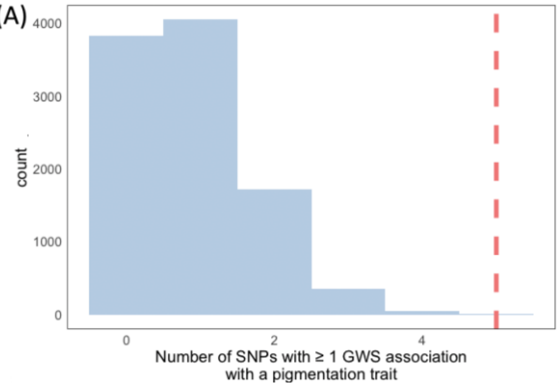

(B)

Number of SNPs with ≥1 GWS association with a pigmentation trait	Number of 13-SNP sets
0	3,829
1	4,046
2	1,720
3	356
4	47
5	2

Figure R. Gene-by-environment interactions (GEI) with skin colour

Mean 25OHD levels (y axis) in individuals with different skin colour (x axis) stratified by rs10832254 (panel A) and rs1352846 (panel B) genotype. 25OHD levels were regressed of confounders and scaled to have a mean 0 and standard deviation of 1 (see Methods). The results of the GEI interaction tests can be found in **Table W in S2 Appendix**.

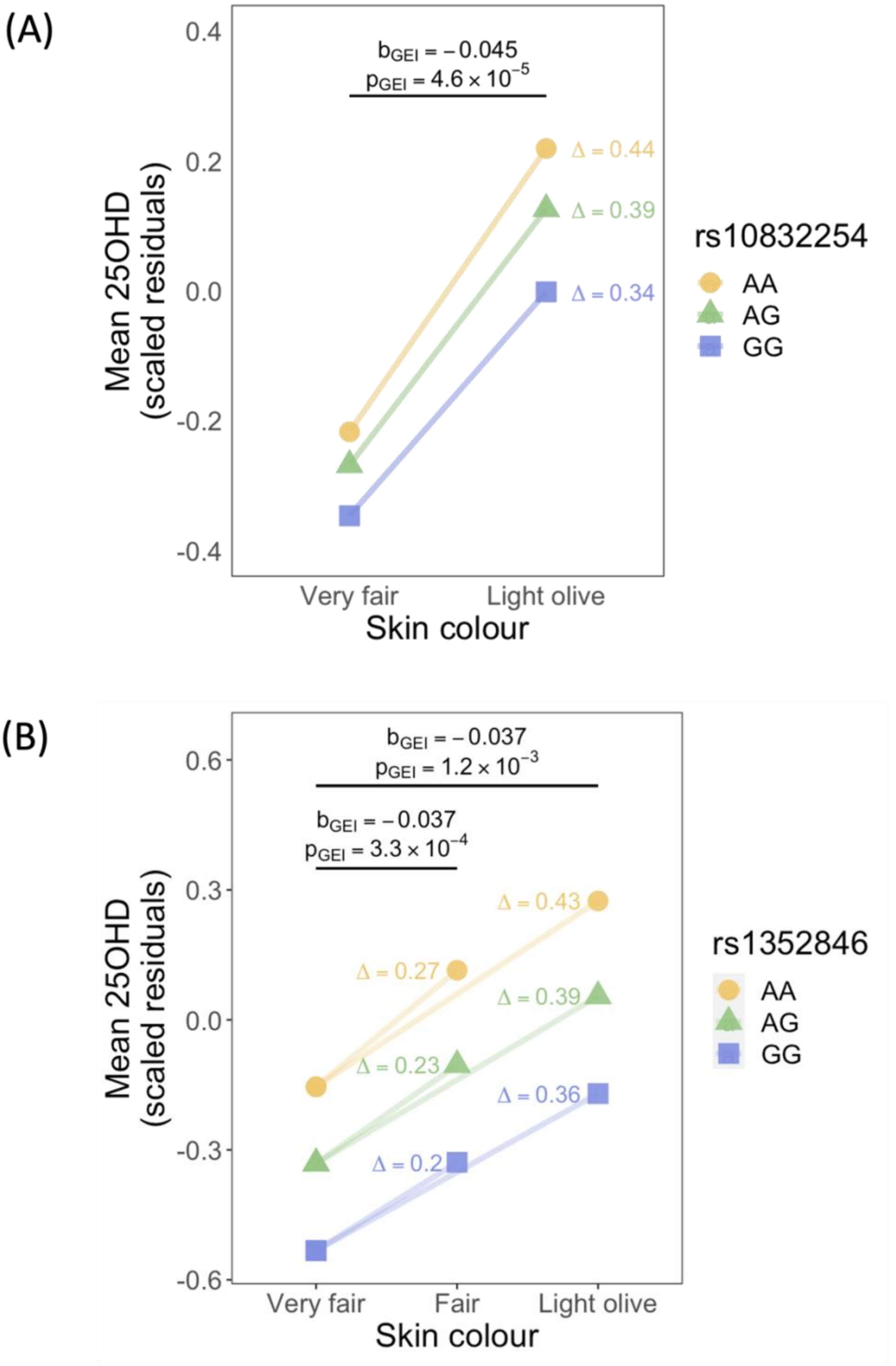

Figure S. Ancestry assignment principal components

Ancestry of UKB participants was genetically inferred based on a principal component (PC) analysis with samples from the 1000 genomes project (1KGP) as reference [27]. This analysis involved two steps. First, PCs were computed for 2,000 participants of the 1KGP that were of European (EUR), South-Asian (SAS), East Asian (EAS) or African (AFR) ancestry. Then, UKB participants were projected onto the 1KGP PCs and assigned to the closest ancestry based on the first three PCs. This method generalizes the k-means method and takes into account the orientation of the reference cluster to improve the clustering. For that reason, in the plots below we see that UKB participants along the same orientation of the 1KGP reference clusters were assigned to those ancestry groups (more details in the Methods). Light colours represent UKB participants. Dark colours represent reference samples from the 1KGP.

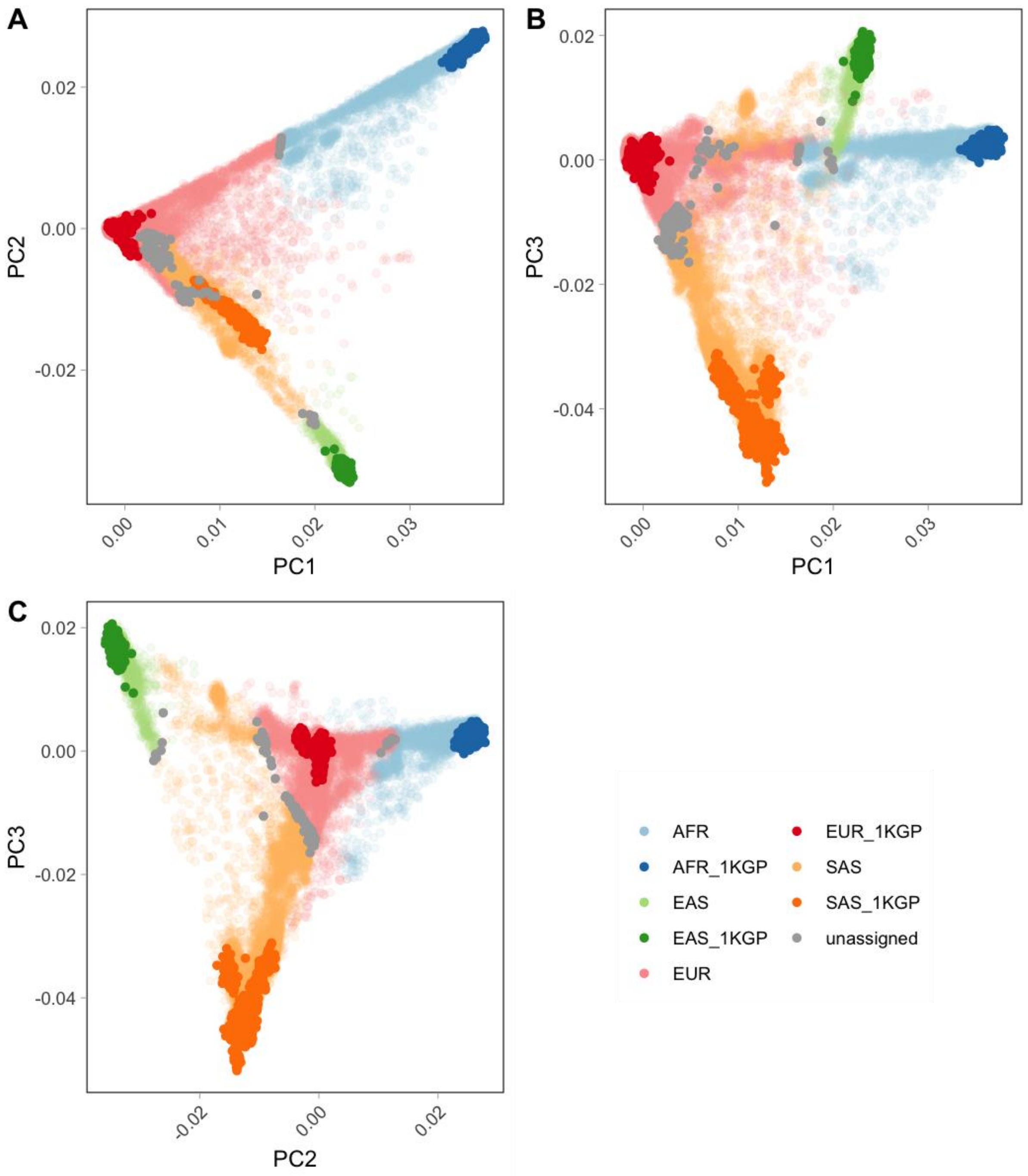

Figure T. European principal components coloured by skin colour

The plot shows the first three principal components computed within the EUR group (wPCs) coloured by skin colour group. The light skin group (N= 324,105) corresponds to participants who reported “Very fair” or “Fair” skin. The dark skin group (N= 85,549) corresponds to participants who reported “Light olive”, “Dark olive” or “Brown” skin. These were the two groups included in the skin-colour-stratified (SCS) analysis. Samples labelled as excluded correspond to EUR participants that had inconsistent self-reports of skin colour across visits (4,492) or that reported black skin (N=38). These individuals were not included in the SCS GWAS. Given the high density of the plot, panel A shows the samples corresponding to light EUR at the back and dark EUR at the front. In panel B the order is inverted.

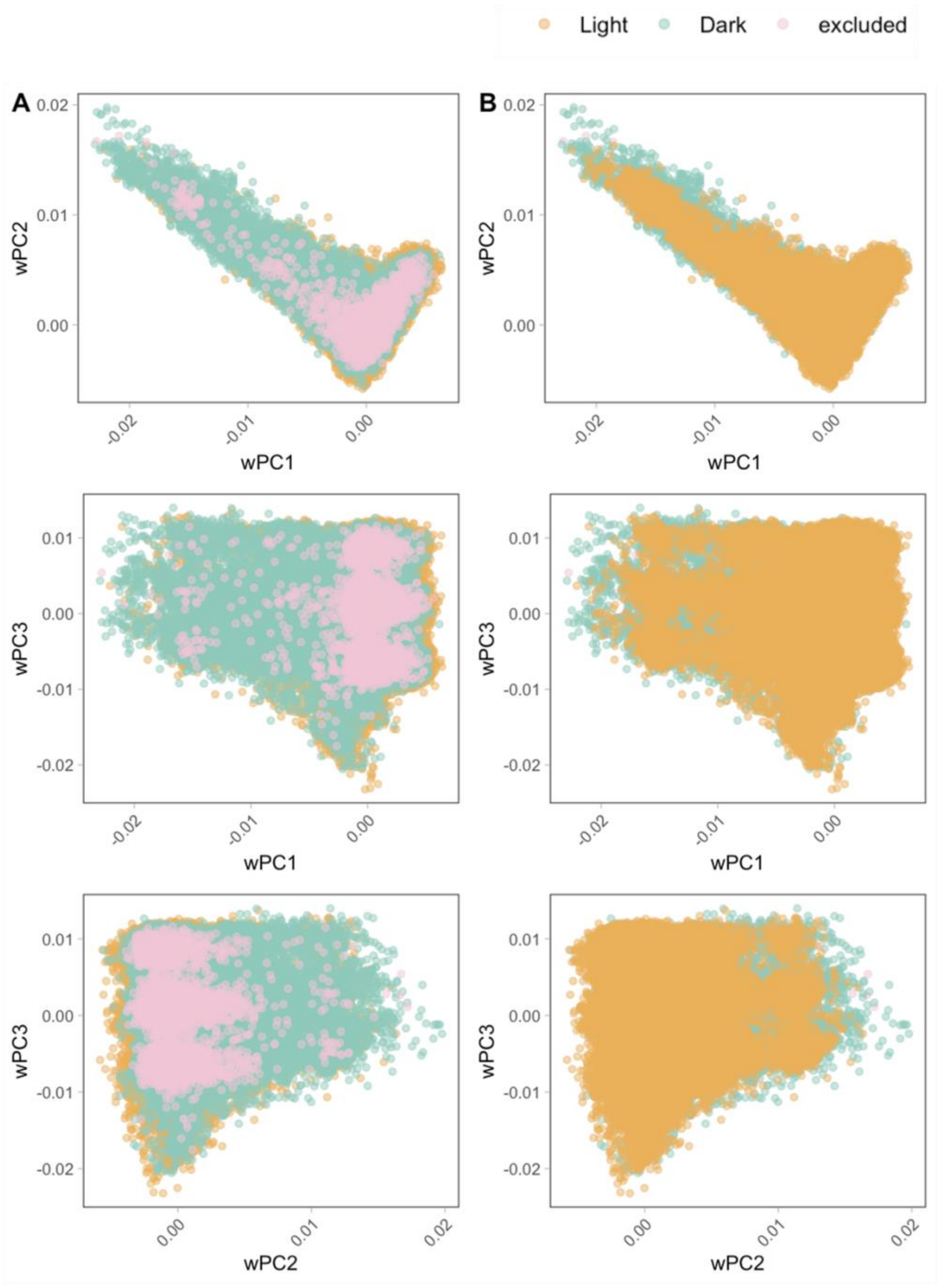

REFERENCES

1. Day FR, Loh PR, Scott RA, Ong KK, Perry JRB. A Robust Example of Collider Bias in a Genetic Association Study. American Journal of Human Genetics. Cell Press; 2016. pp. 392–393. doi:10.1016/j.ajhg.2015.12.019
2. Revez JA, Lin T, Qiao Z, Xue A, Holtz Y, Zhu Z, et al. Genome-wide association study identifies 143 loci associated with 25 hydroxyvitamin D concentration. Nat Commun. 2020;11: 1647. doi:10.1038/s41467-020-15421-7
3. Manousaki D, Mitchell R, Dudding T, Haworth S, Harroud A, Forgetta V, et al. Genome-wide Association Study for Vitamin D Levels Reveals 69 Independent Loci. Am J Hum Genet. 2020;106: 327–337. doi:10.1016/j.ajhg.2020.01.017
4. Jiang X, O'Reilly PF, Aschard H, Hsu YH, Richards JB, Dupuis J, et al. Genome-wide association study in 79,366 European-ancestry individuals informs the genetic architecture of 25-hydroxyvitamin D levels. Nat Commun. 2018;9. doi:10.1038/s41467-017-02662-2
5. Zhu Z, Zheng Z, Zhang F, Wu Y, Trzaskowski M, Maier R, et al. Causal associations between risk factors and common diseases inferred from GWAS summary data. Nat Commun. 2018;9. doi:10.1038/s41467-017-02317-2
6. Yang J, Lee SH, Goddard ME, Visscher PM. GCTA: A tool for genome-wide complex trait analysis. Am J Hum Genet. 2011;88: 76–82. doi:10.1016/j.ajhg.2010.11.011
7. Watanabe K, Stringer S, Frei O, Umićević Mirkov M, de Leeuw C, Polderman TJC, et al. A global overview of pleiotropy and genetic architecture in complex traits. Nat Genet. 2019;51: 1339–1348. doi:10.1038/s41588-019-0481-0
8. Lloyd-Jones LR, Robinson MR, Moser G, Zeng J, Beleza S, Barsh GS, et al. Inference on the genetic basis of eye and skin color in an admixed population via bayesian linear mixed models. Genetics. 2017;206: 1113–1126. doi:10.1534/genetics.116.193383
9. Goddard ME, Hayes BJ, Meuwissen THE. Genomic selection in livestock populations. Genet Res (Camb). 2011/03/23. 2010;92: 413–21. doi:10.1017/S0016672310000613
10. Moser G, Lee SH, Hayes BJ, Goddard ME, Wray NR, Visscher PM. Simultaneous discovery, estimation and prediction analysis of complex traits using a bayesian mixture model. PLoS Genet. 2015;11: e1004969. doi:10.1371/journal.pgen.1004969
11. Yang J, Ferreira T, Morris AP, Medland SE, Madden PAF, Heath AC, et al. Conditional and joint multiple-SNP analysis of GWAS summary statistics identifies additional variants influencing complex traits. Nat Genet. 2012;44: 369–375. doi:10.1038/ng.2213
12. Backman JD, Li AH, Marcketta A, Sun D, Mbatchou J, Kessler MD, et al. Exome sequencing and analysis of 454,787 UK Biobank participants. Nature. 2021;599: 628–634. doi:10.1038/s41586-021-04103-z
13. Ruan Y, Lin Y-F, Feng Y-CA, Chen C-Y, Lam M, Guo Z, et al. Improving polygenic prediction in ancestrally diverse populations. Nat Genet. 2022;54: 573–580. doi:10.1038/s41588-022-01054-7
14. Privé F, Aschard H, Carmi S, Folkersen L, Hoggart C, O'Reilly PF, et al. Portability of 245 polygenic scores when derived from the UK Biobank and applied to 9 ancestry groups from the same cohort. Am J Hum Genet. 2022;109: 12–23. doi:10.1016/j.ajhg.2021.11.008
15. Weir BS, Cockerham CC. ESTIMATING F-STATISTICS FOR THE ANALYSIS OF POPULATION STRUCTURE. Evolution. 1984;38: 1358–1370. doi:10.1111/j.1558-5646.1984.tb05657.x

16. Elhaik E. Empirical distributions of F(ST) from large-scale human polymorphism data. PLoS One. 2012;7: e49837. doi:10.1371/journal.pone.0049837
17. Mersha TB, Abebe T. Self-reported race/ethnicity in the age of genomic research: its potential impact on understanding health disparities. Hum Genomics. 2015;9: 1. doi:10.1186/s40246-014-0023-x
18. Bycroft C, Freeman C, Petkova D, Band G, Elliott LT, Sharp K, et al. The UK Biobank resource with deep phenotyping and genomic data. Nature. 2018;562: 203–209. doi:10.1038/s41586-018-0579-z
19. Using Population Descriptors in Genetics and Genomics Research. Washington, D.C.: National Academies Press; 2023. doi:10.17226/26902
20. Coop G. Genetic similarity versus genetic ancestry groups as sample descriptors in human genetics. 2022. Available: http://arxiv.org/abs/2207.11595
21. Ross AC, Taylor CL, Yaktine AL, Valle HB Del, Review C. Review of Potential Indicators of Adequacy and Selection of Indicators : Calcium and Vitamin D. Proofs. 2010.
22. Lips P, Cashman KD, Lamberg-Allardt C, Bischoff-Ferrari HA, Obermayer-Pietsch B, Bianchi ML, et al. Current vitamin D status in European and Middle East countries and strategies to prevent vitamin D deficiency: a position statement of the European Calcified Tissue Society. Eur J Endocrinol. 2019;180: P23–P54. doi:10.1530/EJE-18-0736
23. Amrein K, Scherkl M, Hoffmann M, Neuwersch-Sommeregger S, Köstenberger M, Tmava Berisha A, et al. Vitamin D deficiency 2.0: an update on the current status worldwide. European Journal of Clinical Nutrition. Eur J Clin Nutr; 2020. pp. 1498–1513. doi:10.1038/s41430-020-0558-y
24. Harris SS. Vitamin D and African Americans. Journal of Nutrition. J Nutr; 2006. pp. 1126–1129. doi:10.1093/jn/136.4.1126
25. Yang J, Benyamin B, McEvoy BP, Gordon S, Henders AK, Nyholt DR, et al. Common SNPs explain a large proportion of the heritability for human height. Nat Genet. 2010;42: 565–569. doi:10.1038/ng.608
26. Jiang L, Zheng Z, Qi T, Kemper KE, Wray NR, Visscher PM, et al. A resource-efficient tool for mixed model association analysis of large-scale data. Nat Genet. 2019;51: 1749–1755. doi:10.1038/s41588-019-0530-8
27. Auton A, Abecasis GR, Altshuler DM, Durbin RM, Bentley DR, Chakravarti A, et al. A global reference for human genetic variation. Nature. 2015;526: 68–74. doi:10.1038/nature15393
